# Investigation of monoterpenoid resistance mechanisms in *Pseudomonas putida* and their consequences for biotransformations

**DOI:** 10.1007/s00253-020-10566-3

**Published:** 2020-04-16

**Authors:** Florence Miramella Schempp, Katharina Elisabeth Hofmann, Jia Mi, Ferdinand Kirchner, Annika Meffert, Hendrik Schewe, Jens Schrader, Markus Buchhaupt

**Affiliations:** 1 DECHEMA-Forschungsinstitut, Industrial Biotechnology, Theodor-Heuss-Allee 25, 60486 Frankfurt am Main, Germany; 2 Faculty Biological Sciences, Goethe University Frankfurt, Max-von-Laue-Str. 9, 60438 Frankfurt am Main, Germany

**Keywords:** *Pseudomonas putida* GS1, Monoterpenes, Monoterpenoids, Resistance, Tolerance, Ttg efflux pumps

## Abstract

Monoterpenoids are widely used in industrial applications, e.g. as active ingredients in pharmaceuticals, in flavor and fragrance compositions, and in agriculture. Severe toxic effects are known for some monoterpenoids making them challenging compounds for biotechnological production processes. Some strains of the bacterium *Pseudomonas putida* show an inherent extraordinarily high tolerance towards solvents including monoterpenoids. An understanding of the underlying factors can help to create suitable strains for monoterpenoids de novo production or conversion. In addition, knowledge about tolerance mechanisms could allow a deeper insight into how bacteria can oppose monoterpenoid containing drugs, like tea tree oil. Within this work, the resistance mechanisms of *P. putida* GS1 were investigated using selected monoterpenoid-hypertolerant mutants. Most of the mutations were found in efflux pump promoter regions or associated transcription factors. Surprisingly, while for the tested monoterpenoid alcohols, ketone, and ether high efflux pump expression increased monoterpenoid tolerance, it reduced the tolerance against geranic acid. However, an increase of geranic acid tolerance could be gained by a mutation in an efflux pump component. It was also found that increased monoterpenoid tolerance can counteract efficient biotransformation ability, indicating the need for a fine-tuned and knowledge-based tolerance improvement for production strain development.

Key points

• *Altered monoterpenoid tolerance mainly related to altered activity of efflux pumps.*

*• Increased tolerance to geranic acid surprisingly caused by decreased export activity.*

*• Reduction of export activity can be beneficial for biotechnological conversions.*

**Electronic supplementary material:**

The online version of this article (10.1007/s00253-020-10566-3) contains supplementary material, which is available to authorized users.

## Introduction

Monoterpenoids are a class of natural products containing more than 1000 different substances (Breitmaier 2005). All contain a linear, cyclic, or bicyclic C_10_ backbone (Habermehl et al. 2008) forming different hydrocarbons or oxygenated compounds (Berger 2007; Schrader and Bohlmann 2015). In nature, monoterpenoids are ubiquitously present in plants, e.g., in essential oils of coniferous wood, in citrus fruits, and in flowers (Schrader 2010). In industry, monoterpenoids are widely used in pharmaceuticals, flavor and fragrance, and agriculture (Habermehl et al. 2008; Chen et al. 2015). However, for many monoterpenoids, the extraction from natural sources poses challenges low concentration in the raw material or because the natural sources are constantly ceasing. Furthermore, many of the structurally more complex terpenoids cannot be chemically synthesized in an economic way. Therefore, microbial production or conversion processes can provide attractive alternatives, particularly if regioselective or stereoselective reactions are included (Berger 2007; Chen et al. 2015).

Monoterpenoids have certain physicochemical characteristics that impede the development of industrial bioprocesses dealing with such compounds as educts and/or products: poor water solubility, high volatility, and distinct cytotoxicity (Trombetta et al. 2005; Berger 2007; Schrader 2010). The latter is related to their accumulation in the cell membrane, leading to an increased membrane fluidity and disturbance of essential cellular functions. In addition, protein denaturation and oxidative damage are caused by incubation of cells with certain monoterpenoids (Andrews et al. 1980; Uribe et al. 1985; Sikkema et al. 1994; Trombetta et al. 2005; Schrader 2010).

It is known that certain microorganisms, among others some strains of *Pseudomonas putida*, can cope with high concentrations of organic solvents (Ramos et al. 2015), including monoterpenoids (Speelmans et al. 1998; Mi et al. 2014). *P. putida* is a gram-negative, saprotrophic soil bacterium (Nelson et al. 2002; Silby et al. 2011) with a diverse catabolism, including the ability to degrade different organic solvents (Timmis 2002; Wu et al. 2011; Ramos et al. 2015). Several *P. putida*-based biotechnological production processes for monoterpenoids have been described, such as the biotransformation of limonene to perillic acid (Speelmans et al. 1998; Mars et al. 2001) or perillyl alcohol (van Beilen et al. 2005; Cornelissen et al. 2011; Cornelissen et al. 2013), the de novo synthesis of geranic acid starting from glycerol (Mi et al. 2014), or the regioselective and stereoselective hydroxylation of 1,8-cineole to (1*R*)-6β-hydroxy-1,8-cineole (Mi et al. 2016).

To further increase the native monoterpenoid tolerance of *P. putida* and to enable the transfer of underlying resistance mechanisms to suitable host strains for biotechnological production processes, the molecular factors of monoterpenoid tolerance and potential specificities for certain structural elements have to be determined. The general solvent tolerance mechanisms in *P. putida* have been studied intensively with a focus on the model compounds toluene or butanol, reviewed previously (Isken and de Bont 1998; Ramos et al. 2002; Ramos et al. 2015). The multifactorial response after solvent exposure involves changes in membrane composition (Pinkart and White 1997; Ramos et al. 1997; Isken and de Bont 1998; Heipieper et al. 2003), activation of a general stress response system, enhanced energy generation (Ramos et al. 2015), and induction of specific efflux pumps that extrude solvents to the medium (Isken and de Bont 1996; Ramos et al. 1997; Kieboom et al. 1998). In *P. putida* DOT-T1E, three RND (resistance-nodulation-division) efflux systems have been identified to be directly involved in solvent tolerance (Ramos et al. 2015). These efflux systems (TtgABC, TtgDEF, and TtgGHI) are encoded by the so-called toluene tolerance genes (Ramos et al. 1998; Mosqueda and Ramos 2000; Duque et al. 2001; Rodríguez-Herva et al. 2007). All three efflux pumps consist of three components: an inner membrane protein (TtgB/E/H), which binds the substrates and acts as the extrusion element, an outer membrane protein that reaches into the periplasmic space to form a channel (TtgC/F/I), and a lipoprotein that plays a role in stabilizing the interaction between the other two elements (TtgA/D/G) (Ramos et al. 2002). The Ttg pump-mediated efflux is energized by the proton motive force across the cytoplasmic membrane (Udaondo et al. 2012; Ramos et al. 2015). Expression of the *ttg* operons is repressed by the transcription factors TtgR, TtgT, and TtgV, which are encoded adjacent to the *ttg* efflux pump operons (Duque et al. 2001; Ramos et al. 2002; Rojas et al. 2003; Teran et al. 2003; Ramos et al. 2005; Krell et al. 2007; Terán et al. 2007; Ramos et al. 2015). In other *Pseudomonas* strains, similar tolerance mechanisms have been described, such as the SrpABC efflux pump in *P. putida* S12 (Isken and de Bont 1996; Kieboom et al. 1998) or the Mex efflux systems in *Pseudomonas aeruginosa* (Poole et al. 1993; Gotoh et al. 1995; Poole et al. 1996). Whether these general solvent resistance mechanisms of *P. putida* also apply to monoterpenes and monoterpenoids remains to be investigated.

In vitro studies by Sikkema et al. (1994) and experiments with *Escherichia coli* by Trombetta et al. (2005) have shown that the toxic effect of monoterpenes and their derivatives is at least partially caused by the incorporation of the substances into the bacterial cell membranes, whereby the function of the membranes is disturbed. In addition, they probably also penetrate the cells and interact with other targets, such as enzymes (Sikkema et al. 1994; Trombetta et al. 2005). With regard to tolerance mechanisms, the MexAB-OprM efflux pump of *P. aeruginosa* was shown to play a decisive role for the tolerance of this species to tea tree oil and its monoterpenoid compounds (Papadopoulos et al. 2008). The Mex efflux systems of *P. aeruginosa* are very similar to the Ttg and Srp efflux pumps of *P. putida* (Ramos et al. 2002). In addition, other studies with *E. coli* revealed that active efflux of monoterpenoids is an important factor for increased tolerance (Dunlop et al. 2011). These studies let us expect an involvement of *P. putida* efflux systems in monoterpenoid tolerance.

To test this hypothesis and to identify and further characterize mechanisms specifically responsible for the natural high tolerance of *P. putida* GS1 towards several monoterpenoids, we selected monoterpenoid-hypertolerant mutants and characterized them via genome sequencing, deletion and complementation experiments, tolerance assays, qPCRs, and efflux activity measurements. Moreover, the impact of increased monoterpenoid tolerance on a biotechnological production process was investigated.

## Materials and methods

### Chemicals and media

All chemicals were purchased from Sigma-Aldrich (Taufkirchen, Germany), Carl-Roth (Karlsruhe, Germany), or Merck Millipore (Darmstadt, Germany).

Monoterpenoids were acquired with different purities: 1,8-cineole (≥ 99%), citral (≥ 98%), geranic acid (≥ 85%), geraniol (≥ 99%), geranyl acetate (≥ 99%), geranyl formate (≥ 95%), linalool (≥ 97%), myrcene (≥ 95%), α**-**terpinene (≥ 95%), γ-terpinene (≥ 98.5%), (+)-terpinen-4-ol (≥ 98.5%), (+)-α-terpineol (≥ 97%), α**-**terpinyl acetate (≥ 97%), and (1*S*)-(−)-verbenone (≥ 99%).

For cultivation of *E. coli* and *P. putida*, lysogeny broth (LB) or terrific broth (TB) medium was used. For solid media, 17 g l^−1^ agar-agar was added. Antibiotics and other supplements were used at the following concentrations, if required: kanamycin (Km) 50 μg ml^−1^, gentamicin (Gm) 25 μg ml^−1^, tetracycline (Tet) 50–150 μg ml^−1^, and L-rhamnose 2 mg ml^−1^.

### Strains, plasmids, and oligonucleotides

All strains, plasmids, and oligonucleotides used in this study are listed in Online Resource Table S1.

*P. putida* cells were routinely grown at 30 °C and *E. coli* cells at 37 °C. All oligonucleotides were ordered from Sigma-Aldrich (Taufkirchen, Germany).

### Strain and plasmid construction

Deletion mutants of *ttgR*, *ttgT*, and 10 nucleotides of *ttgABC*-5′-UTR were obtained following the method published by Martínez-García and de Lorenzo (2011). For this purpose, plasmids pEMG-Δ*ttgR*, pEMG-Δ*ttgT*, and pEMG-Δ10 nt-*ttgABC* were constructed using primer P1–P4, P5–P8, or P9–P12, respectively. The correct deletion was checked by colony PCR and sequencing of the obtained PCR fragments.

Plasmids pMiS4-*ttgR* and pMiS4-*ttgT* were constructed by amplifying the *ttgR* and the *ttgT* genes together with their native promoter regions via PCR using *P. putida* GS1 gDNA as template and primer P13 and P14 or P15 and P16, respectively.

All plasmids used in this study were constructed using Gibson isothermal assembly (Gibson et al. 2009) and chemical competent *E. coli* cells (Inoue et al. 1990). The correct and sequenced plasmids were electroporated into *P. putida* GS1 strains following a protocol by Choi et al. (2006).

### Generation of mutant library

Transposon mutagenesis of *P. putida* GS1 was performed following methods published by Klebensberger et al. (2007) and Li et al. (2010). Introduction of plasmid pALMAR-3, harboring a *Himar1 mariner* transposon (Lampe et al. 1999), into *P. putida* GS1 was performed by bi-parental mating with *E. coli* S17-1 λpir as donor. For *E. coli* S17-1 λpir harboring pALMAR-3, 20 ml LB medium containing 30 μg ml^−1^ kanamycin was inoculated with an OD_600_ of 0.1 from an overnight pre-culture. For *P. putida* GS1, 100 ml LB medium was inoculated with an OD_600_ of 0.05. After reaching OD_600_ of 2 or 0.3, respectively, 8 ml of LB culture of the *E. coli* donor strain and 100 ml of the recipient strain *P. putida* GS1 were harvested, washed twice with 8 ml pre-warmed LB medium, and resuspended in 400 μl pre-warmed LB medium each. Subsequently, the cell suspensions were mixed in a 1:1 volume ratio (cell ratio *E. coli* to *P. putida* around 1:2) and placed on LB agar. After incubation for 24 h at 30 °C, the cells were resuspended from the plate with 2 ml 0.9% (*w*/*v*) NaCl solution. One hundred microliter aliquots of the cell suspension were spread on *Pseudomonas* isolation agar plates (King et al. 1954) containing 150 μg ml^−1^ tetracycline to select for *P. putida* transposon mutants. After incubation for 24 h at 30 °C, colonies were washed from the plates with 2 ml LB medium containing 20% glycerol and stored at − 80 °C.

### Growth selection of monoterpenoid-hypertolerant mutants

For growth selection under different monoterpenoid stress conditions, both *P. putida* GS1 wildtype (WT) cells and the transposon mutant library were used as a mixture to ensure large cell variety at the starting point. The monoterpenoids were added directly to the bacteria cultures in different, partly increasing concentrations: 1,8-cineole 20, 40, and 60 mM; geranic acid 35 and 90 mM; geraniol 32 and 65 mM; α-terpineol 17.5 mM; and verbenone 35 mM. The cryo stocks were thawed on ice and grown overnight at 30 °C in shake flasks with 20 ml TB medium containing 50 μg ml^−1^ tetracycline.

For the selection of 1,8-cineole-hypertolerant mutants, the preculture was diluted to an OD_600_ of 0.1 with TB medium. The cells were cultivated with the stressor for 24 h at 30 °C in a microbioreactor system (BioLector®) at 1000 rpm using 1.5 ml culture volume in 48-well Flowerplates® (m2p-labs GmbH, Baesweiler, Germany) covered by gas-permeable sealing foil. From the stationary phase culture, new TB cultures were inoculated to an OD_600_ of 0.1 and again cultivated in the microbioreactor system with 1,8-cineole. This procedure was followed for five cultivation steps. After each round, cultures with improved tolerance properties were selected for the next selection round. Each culture was cultivated in triplicates. Only for the first cultivation 50 μg ml^−1^ tetracycline was added to the medium. After cultivation 2, 3, and 4, the cells were spread on LB-agar plates and incubated over night at 30 °C or for 3 days at room temperature. Subsequently, the cells were washed from the agar plates with 3 ml NaCl (0.9%), and 1 ml of the cell suspension was used to inoculate the new culture.

For the selection of geranic acid, geraniol, α-terpineol, and verbenone-hypertolerant mutants 200 μl preculture was transferred into 20 ml TB medium each and the respective stressor was added. The cultures were incubated in shake flasks for around 24 h at 30 °C. Between five and seven sequential cultivation rounds were conducted for each monoterpenoid. For each further round, the main culture was inoculated to a start OD_600_ of 0.1. Before the last cultivation round for all monoterpenoids, the cells were spread out on LB agar to obtain single colonies for inoculation.

Based on the growth performance of the different mutants in the last cultivation round, for each selection substance one (or in the case of verbenone two) candidate strains were selected for further characterization.

### Genetic characterization of monoterpenoid-hypertolerant mutants

In order to determine the genotype of the selected mutant strains, the mutation sites were mapped via splinkerette PCR (Devon et al. 1995; Mikkers et al. 2002) (CR mutant) or genome sequencing (TR, GR, GAR, VR1, VR2 mutant).

For the 1,8-cineole selected mutant (CR), gDNA was extracted using the *GenElute Bacterial Genomic DNA* kit (Sigma-Aldrich, Taufkirchen, Germany). Of total gDNA, 1.5 μg was digested with BamHI. Subsequently, hybridized splinkerette adapter (P17 and P18, 1.2 pmol) was ligated to 300 ng of the DNA fragments using T4-Ligase (NEB, Frankfurt am Main, Germany). Ligation products were isolated using the *DNA Clean & Concentrator™-5* kit (Zymo Research, Freiburg, Germany). Transposon-chromosome junctions were amplified by PCR with a primer specific for the adapter (P19) and a primer specific for the mariner transposon (P20). After purification of the PCR product with the *DNA Clean & Concentrator™-5* kit (Zymo Research, Freiburg, Germany), a second PCR with the first PCR product as template using primer P21 and P22 was conducted. Finally, the obtained PCR product was purified as described before and sequenced.

To localize the transposon integration site, the sequences adjacent to the transposon were mapped to the genome sequence of *P. putida* GS1. The latter was provided by the company GenXPro (Frankfurt am Main, Germany) using the SMRT method and with the annotation software Prokka (v1.11) (Torsten Seemann 2014).

For the genotypic characterization of the mutants GAR, GR, TR, VR1, and VR2, for each strain, 200 ml TB medium was inoculated to an OD_600_ of 0.2 from an overnight culture and incubated at 30 °C and 180 rpm. When cultures reached an OD_600_ between 2.5 and 5, cells were harvested, washed with 20 ml H_2_O, and frozen with liquid nitrogen to store at − 80 °C. Genomic DNA preparation and genome sequencing via SBS (*sequencing by synthesis*)-Illumina approach (Illumina, San Diego, USA) was conducted by the company GenXPro (Frankfurt am Main, Germany). Reads obtained from sequencing were mapped against the *P. putida* GS1 genome using the software G*eneious* (Biomatters Ltd., Auckland, New Zealand). In order to reduce errors occurring from the sequencing method, the *P. putida* GS1 WT genome was resequenced in parallel with the Illumina approach.

### Monoterpenoid tolerance assays

Pre-cultures of *P. putida* GS1 WT and mutant strains grown in TB medium were used to inoculate TB cultures to an OD_600_ of 0.1, supplemented with appropriate antibiotics if required. The respective monoterpenoid was added directly after inoculation. The concentrations tested for each compound, without consideration of purity, were 1,8-cineole 40, 60, 100, and 200 mM; citral 25 and 100 mM; geranic acid 100 and 200 mM of substance with 85% geranic acid; geraniol 40, 65, and 100 mM; geranyl acetate 25 and 100 mM; geranyl formate 25 and 100 mM; linalool 25 and 100 mM; myrcene 17.5, 35, 60, 100, and 200 mM; α-terpinene 25 and 200 mM; γ-terpinene 17.5, 35, 60, 100, and 200 mM; (+)-terpinen-4-ol 80 mM; (+)-α-terpineol 17.5, 35, and 60 mM; α-terpinyl acetate 25 and 100 mM; (1*S*)-(−)-verbenone 15, 25, and 35 mM.

Cells exposed to the different chemicals were incubated for 48 h at 30 °C in a microbioreactor system (BioLector®) at 1000 rpm and 85% humidity using 1 ml culture volume in 48-well Flowerplates® (m2p-labs GmbH) covered with gas-permeable sealing foil. Biomass formation was monitored via scattered light signal intensity at 620 nm. If the strains contained the pMiS4-eGFP plasmid, growth was monitored additionally via GFP fluorescence signal intensity, using an excitation filter of 488 nm and an emission filter of 520 nm. For induction of GFP expression, L-rhamnose was added to a final concentration of 0.2% (*w*/*v*) directly after inoculation. All growth comparison experiments in Flowerplates® were performed at least in triplicates. Culture samples were distributed randomly on the plate.

For quantification of verbenone concentration in the cell suspensions and in cell-free medium over time, cells were cultivated as described above but with 1.5 ml culture volume. Verbenone (35 mM) was added at inoculation. Samples of 180 μl were taken at time points *t* = 0, 15, 22, 40, and 45 h. To the samples, 20 μl 1 M H_2_SO_4_ was added and analytes were extracted using 200 μl ethyl acetate. Samples were centrifuged (16,000*×g*, 2 min), and the organic phase was analyzed by GC-MS (GC-17A with QP5050A detector, Shimadzu) with a VB-5 column (30 m × 0.25 mm × 0.25 μm, ValcoBond® (Valco Instruments Co. Inc. and VICI AG)). Measurements were conducted as follows: helium as carrier gas, split ratio 35, injections at 250 °C, and a column flow of 2.6 ml min^−1^. The column temperature was programmed as follows: 80 °C for 3 min, 7 °C min^−1^ up to 150 °C followed by 150 °C for 2 min. Absolute concentration of verbenone was calculated from chromatogram peak areas by comparison to a calibration curve prepared by measuring a dilution series of verbenone standard with known concentrations.

### RNA extraction, cDNA synthesis, and quantitative polymerase chain reaction

In order to quantify differences in expression of the efflux pump genes between *P. putida* GS1 WT and mutants, total RNA was harvested from growing cultures following a protocol modified from Otto et al. (2019). Therefore, pre-cultures grown in TB medium were used to inoculate TB medium to an OD_600_ of 0.1. Cultures were cultivated at 30 °C and 200 rpm until mid-exponential growth phase (OD_600_ 0.9–1.1). Samples of 1 ml cell suspension were harvested (13,000*×g*, 2 min), resuspended in 800 μl *RNA/DNAShield* solution (Zymo Research Europe GmbH), and stored at − 80 °C until further analysis. RNA was extracted from cells using *Quick RNA™ Miniprep Plus Kit* (Zymo Research Europe GmbH) following manufacturer’s instructions including in-column DNase treatment. cDNA was prepared from purified RNA using the *IScript™ gDNA Clear cDNA Synthesis Kit* (Bio-Rad Laboratories, Inc.). The expression levels of different efflux pump genes were analyzed using primers designed by an online Realtime PCR tool (http://www.idtdna.com/scitools/Applications/RealTimePCR). Primers are listed in Table S1 (P23–34). As reference gene, *rpoD* was used and amplified with primers described previously (Franden et al. 2018). Quantitative PCR was performed using *QuantiTec SYBR Green PCR Kit* (Qiagen) on a *PikoReal™ Real-Time PCR* System (Thermo Scientific). The reaction conditions were 15 min at 95 °C, 45× (15 s at 94 °C, 30 s at 60 °C, and 30 s at 72 °C), followed by melting curve analysis (30 s starting at 50 °C, increasing 0.2 °C per cycle, ending at 95 °C). Experiments were performed with biological triplicates. No-template controls were run for every primer pair, and no-RT (reverse transcriptase) controls were run for every RNA sample. Transcript levels of *ttg* genes were estimated by comparing their Ct (cycle threshold) values to the Ct value of the housekeeping gene *rpoD* (Wang and Nomura 2010)*.* Final expression levels were averaged for each mutant strain and normalized to the expression level of the *P. putida* GS1 wild-type strain using the following formula:

ΔΔCt = ΔCtE − ΔCtC

with ΔCtE = Ct (*ttg* gene mutant) − Ct (*rpoD* gene mutant).

and ΔCtC = Ct (*ttg* gene wild type) − Ct (*rpoD* gene wild type).

Gene expression fold change was calculated as follows: Fold change = 2^−ΔΔCt^.

For statistical analysis, Shapiro-Wilk normality test, followed by pairwise comparison using van der Waerden normal score test with Benjamini-Yakutiel *p* value adjustment, was applied on ΔCt values.

### Efflux activity assay

Efflux pump activity of *P. putida* wild type and mutants was determined via resazurin accumulation assay described by Vidal-Aroca et al. (2009). Ten milliliter LB medium in 100-ml Erlenmeyer flasks was inoculated from overnight cultures to an OD_600_ of 0.1 and grown at 30 °C and 180 rpm to mid-exponential phase (OD_600_ around 1). Cells were harvested by centrifugation (12,000*×g*, 3 min). The cell pellets were washed two times with 1× PBS buffer and resuspended in PBS + 0.4% glucose. Fluorescence signal was measured with an *Infinite® 200 PRO* microtiter plate reader (Tecan). Experiments were performed with biological triplicates. The slope of the fluorescence increase was averaged for each strain over 50 min of the experiment and compared to the wild-type value. For statistical analysis, Shapiro-Wilk normality test, followed by pairwise comparison using van der Waerden normal score test with Benjamini-Yakutiel *p* value adjustment, was applied on slope mean values of fluorescence measurements.

### Biotransformation experiments

An approach modified from Mi et al. (2014) was used. Pre-cultures of *P. putida* GS1 WT and mutant strains grown in TB medium were diluted with TB medium to an OD_600_ of 0.1. Cultures were divided into 1.5 ml aliquots in 48-well Flowerplates® (m2p-labs GmbH, Baesweiler, Germany), and 35 mM geraniol as biotransformation substrate was added directly after inoculation. Cells were incubated for 38 h at 30 °C in a microbioreactor system (BioLector®) at 1000 rpm covered with gas-permeable sealing foil. Biomass formation was monitored via scattered light signal intensity at 620 nm. Geranic acid concentrations at different time points (0, 12, 21, and 38 h) were determined by HPLC analysis. All strains were tested in triplicates, distributed randomly on the plate.

To *P. putida* culture samples of 150 μl, 15 μl 1 M HCl was added and analytes were extracted using 165 μl hexane containing 1 mM thymol as an internal standard. Samples were centrifuged (16,000*×g*, 5 min), and the organic phase was analyzed by HPLC, consisting of a diode array detector and a C18 column (Alltech Alltima, C18, 5 μm, 250 × 4.6 mm; C18 Precolumn, Grace GmbH and Co. KG, Worms, Germany). Substances were separated isocratically using acetonitrile/acidified water (containing 0.05% (*v*/*v*) 3 M phosphoric acid) in a ratio of 45:55 (*v*/*v*) as mobile phase. After each run, the column was washed with 90:10 mixture of acetonitrile/acidified water.

For statistical analysis, Shapiro-Wilk normality test, followed by pairwise comparison using van der Waerden normal score test with Benjamini-Yakutiel *p* value adjustment, was applied.

## Results

### Tolerance of *P. putida* GS1 towards monoterpenoids

**Fig. 1:**
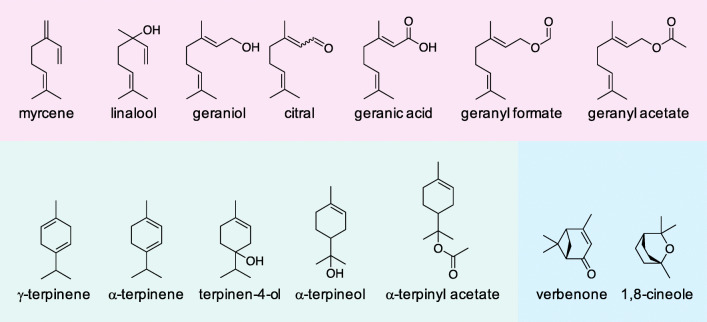
Chemical structures of monoterpenes and monoterpenoids used in this study. Red: linear, green: monocyclic, blue: bicyclic structures

For the development of microbial monoterpenoid production strains, a deep molecular understanding of the tolerance mechanisms for such compounds represents a prerequisite. As *P. putida* shows strong monoterpenoid tolerance (Inoue and Horikoshi 1991; Speelmans et al. 1998; Mi et al. 2014; Rau et al. 2016) and its organic solvent resistance mechanisms have been extensively studied (Isken and de Bont 1998; Ramos et al. 2015), we chose the *P. putida* strain GS1 (Speelmans et al. 1998) to explore its behavior after exposure to a number of different monoterpenoids (Fig. 1).

The effects of the different compounds on the growth of *P. putida* GS1 were investigated by comparing characteristics of the growth curves from microbioreactor cultivations under the influence of exogenously added chemicals. Cell density was monitored via scattered light signal and GFP fluorescence measurements. Experiments without addition and with different concentrations of monoterpenes and monoterpenoids had previously confirmed that growth curves obtained by scattered light signal measurements and fluorescence measurements are comparable for most of the substances tested (Online Resource Fig. S1–Fig. S4) and that GFP gene expression does not influence the growth of *P. putida*. Only in the case of geranic acid an interference of the substance with the scattered light signal could be observed and the GFP signal was necessary to monitor growth.

**Fig. 2:**
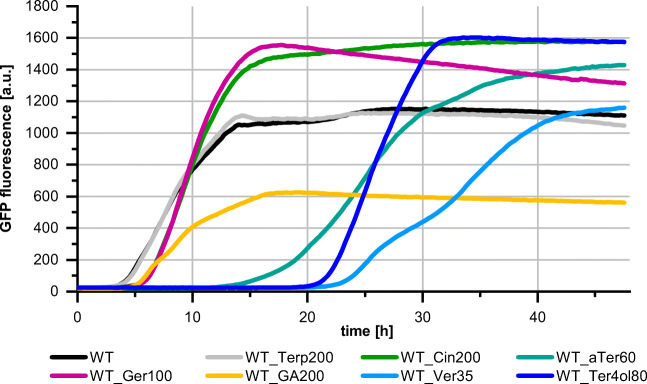
Growth of *P. putida* GS1 (WT) + pMiS4-eGFP without and in the presence of different monoterpenes and monoterpenoids. Terp200: γ-terpinene (200 mM), Cin200: 1,8-cineole (200 mM), aTer60: α-terpineol (60 mM), Ger100: geraniol (100 mM), GA200: geranic acid (200 mM), Ver35: verbenone (35 mM), Ter4ol80: terpinen-4-ol (80 mM). Tolerance assays were conducted in a microbioreactor system over 48 h. Biomass formation was monitored every 10–15 min via GFP fluorescence signal intensity (excitation filter 488 nm; emission filter 520 nm). The data points represent the mean values of three biological replicates. For variations between the replicates of each strain, see Online Resource Fig. S18

Analysis of the resulting data showed that the monoterpenes and monoterpenoids affect growth of *P. putida* GS1 differently (Fig. 2, Online Resource Fig. S2–Fig. S4). The bacterium could cope with the hydrocarbons (myrcene, α-terpinene, γ-terpinene) up to the highest tested concentration of 200 mM and the esterified monoterpenoids (geranyl acetate, geranyl formate, α-terpinyl acetate) up to the highest tested concentration of 100 mM without any apparent effect on its growth. The aldehyde citral, the ether 1,8-cineole, and the alcohols geraniol and linalool caused a slight prolongation of the lag phase when added in concentrations between 100 and 200 mM. However, if geraniol, linalool, or geranyl acetate were added, *P. putida* GS1 reached higher maximal GFP fluorescence intensities compared with cultures without monoterpenoid addition. Addition of 200 mM geranic acid caused a reduction in growth rate and a slightly prolonged lag phase. The most pronounced growth inhibition was observed for the ketone verbenone and the alcohols α**-**terpineol and terpinen-4-ol, which caused lag phase prolongations of more than 10 h if present in concentrations of 35, 60, and 80 mM, respectively.

Testing different concentrations of the same monoterpenoid revealed a positive correlation between compound concentration and severity of growth inhibition (Online Resource Fig. S1).

### Isolation and investigation of monoterpenoid-hypertolerant mutants

In order to investigate the molecular mechanisms of monoterpenoid tolerance in *P. putida* GS1 and their specificities, a mutant library was created by transposon mutagenesis with a *Himar1 mariner* transposon (Lampe et al. 1999; Klebensberger et al. 2007; Li et al. 2010). The mutant library was grown in the presence of one of the toxic compounds 1,8-cineole, geranic acid, geraniol, α**-**terpineol, or verbenone, and monoterpenoid-hypertolerant mutants were selected. To ensure a large diversity at the starting point, also *P. putida* GS1 wild-type cells were added to the mutant selection process. After testing the phenotypes of several mutants, one representative with improved growth characteristics towards the specific selection monoterpenoid (shorter lag phase and/or increased growth rate) compared with the WT strain was chosen for further studies. The strains were named according to the monoterpenoid used as selective agent: 1,8-cineole-resistant (CR), geranic acid-resistant (GAR), geraniol-resistant (GR), α-terpineol-resistant (TR), and verbenone-resistant (VR1) mutant.

**Fig. 3:**
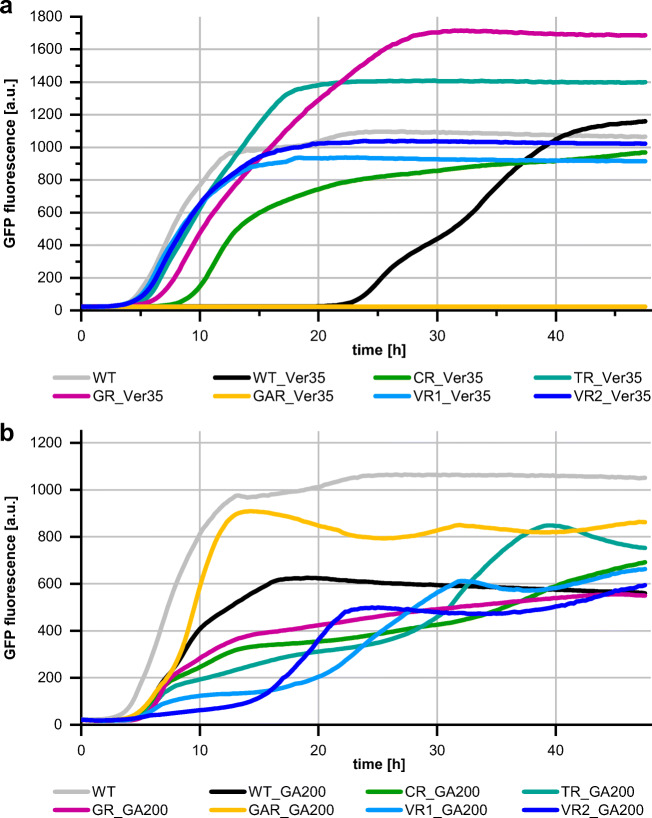
Growth of *P. putida* GS1 WT and mutants + pMiS4-eGFP in the presence of **a** 35 mM verbenone (Ver35) or **b** 200 mM geranic acid (GA200). Tolerance assays were conducted in a microbioreactor system over 48 h. Biomass formation was monitored every 10–15 min via GFP fluorescence signal intensity (excitation filter 488 nm; emission filter 520 nm). The data points represent the mean values of three biological replicas. For variations between the replicas of each strain, see Online Resource Fig. S24 and Fig. S28

Without the addition of monoterpenoids, the mutants showed similar growth curve characteristics as the wild-type strain (Online Resource Fig. S5). Monoterpenoid tolerance assays in microbioreactor cultivations with 1,8-cineole (200 mM), geraniol (100 mM), α-terpineol (60 mM), terpinen-4-ol (80 mM), or verbenone (35 mM) revealed improved tolerance of the mutant strains CR, GR, TR, and VR1 compared with the wild-type strain (Fig. 3a, Online Resource Fig. S6–Fig. S12). The most profound tolerance phenotypes for these compounds were obtained for the TR and the VR1 mutant. In contrary, when CR, GR, TR, and VR1 were exposed to 200 mM geranic acid, their growth was impaired to a higher extent than that of the wild-type strain (Fig. 3b). The only exception was the GAR mutant, which showed an improved tolerance towards geranic acid compared with the wild-type strain (Fig. 3b). However, the GAR strain was much more sensitive towards all other monoterpenoids tested (Fig. 3a, Online Resource Fig. S6–Fig. S12).

**Fig. 4:**
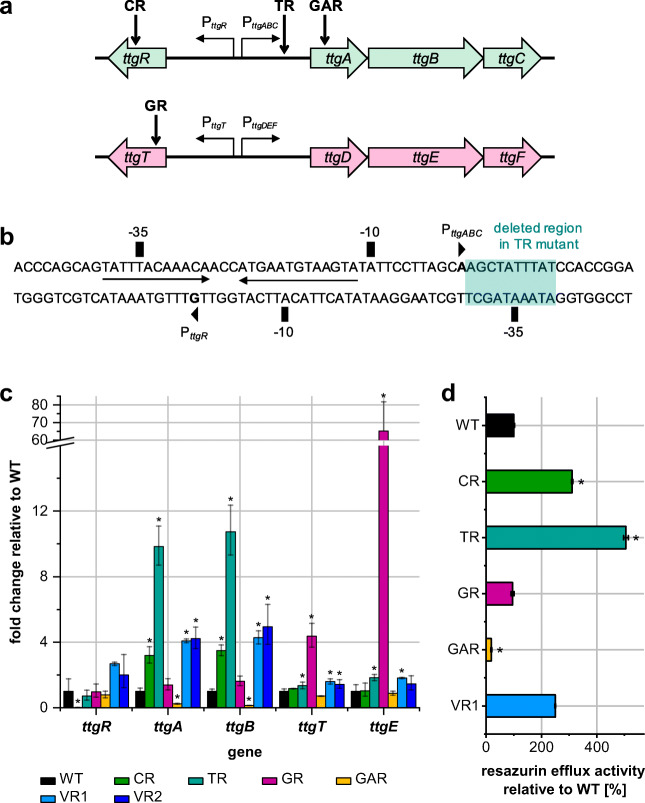
Further characterization of mutant strains (CR, TR, GR, GAR, VR1, and VR2). **a** Localization of identified mutations in different mutant strains. **b** Nucleotide sequence of the *ttgABC-ttgR* intergenic region. Deleted region in TR mutant is highlighted in green. The putative TtgR palindromic recognition site is indicated by the arrows; *ttgABC* and *ttgR* + 1 (arrowheads), − 10, and − 35 points are marked according to Terán et al. (2003). **c** Relative expression levels of *ttg* genes in mutants compared with *P. putida* GS1 wild type (2^−ΔΔCt^). **d** Resazurin efflux pump activity of *P. putida* GS1 mutant strains relative to wild type. Slope mean values of fluorescence increase over time of mutant strains normalized to wild type are given. Cells were grown in LB medium until mid-exponential phase and resuspended in PBS buffer with resazurin. Efflux activity was monitored by measuring the fluorescence intensity of resazurin reduction product resorufin (excitation filter 530 nm; emission filter 590 nm). While a slow increase in fluorescence indicates high efflux activity, a high slope shows low efflux activity. In **c** und **d**, results represent the mean values and standard deviations of three biological replicates. The asterisks indicate a significant difference for the mutant strains compared with wild-type GS1 according to van der Waerden normal score test with Benjamini-Yakutiel *p* value adjustment (*p* < 0.05)

To identify the genotypic characteristics causing the specific monoterpenoid-hypertolerant phenotypes of the mutants, either a splinkerette PCR approach was applied to identify transposon integration sites, or whole genome sequencing. The results are summarized in Fig. 4a, b, Table 1, and Online Resource Table S2. In the CR mutant, the transposon was localized in the *ttgR* gene, the transcriptional regulator of the genes encoding the TtgABC efflux pump. The transposon in the GR mutant was found to be inserted in the *ttgT* gene, the transcriptional regulator repressing transcription of the *ttgDEF* operon. Genome analysis of GAR, TR, and VR1 revealed no mariner transposon sequence but distinct other mutations. In the TR mutant, a 10-nucleotide deletion was identified in the region directly behind the transcription start of the *ttgABC* operon. The deleted region includes the − 35 region of the *ttgR* promoter.

**Table 1:** Mutant strains, selection substances, and identified mutations presumably causing the altered monoterpenoid tolerance phenotypes

Mutant	Selection substance	Mutation responsible for or involved in evolution of hypertolerance phenotype
CR	1,8-Cineole	Transposon insertion in *ttgR* gene (487 nucleotides downstream of start codon) *ttgR* ➔ transcriptional regulator of *ttgABC* operon
TR	α**-**Terpineol	Deletion of 10 nucleotides in 5′-UTR of *ttgABC* mRNA (directly after P_*ttgABC*_ + 1) and − 35 region of P_*ttgR*_
GR	Geraniol	Transposon insertion in *ttgT* gene (40 nucleotides downstream of start codon) *ttgT* ➔ transcriptional regulator of *ttgDEF* operon
GAR	Geranic acid	Deletion of 41 nucleotides in *ttgA* gene (109 nucleotides downstream of start codon) *ttgA* ➔ subunit of TtgABC efflux system
VR1	Verbenone	• 33 different mutations (16-nucleotide deletion, 32 single nucleotide exchanges) • DNA repair protein (*mutS*) • Subunit of respiratory chain protein (*nuoB*)
VR2	Verbenone	• 32 different mutations (1 nucleotide deletion, 1 nucleotide insertion, 30 single nucleotide exchanges) • DNA repair protein (*mutL*) • Subunit of respiratory chain protein (*nuoG*)

In order to verify the correlation between the identified Ttg-related mutations in the CR, GR, and TR mutant and improved monoterpenoid tolerance, *P. putida* GS1 strains were constructed which contain a deletion of either the *ttgR* or the *ttgT* gene or lack the 10 nucleotides in the region between the *ttgR* and *ttgA* open reading frames according to the respective mutation observed in the TR genome. Monoterpenoid tolerance assays conducted with the constructed deletion mutants Δ*ttgR*, Δ*ttgT*, and Δ10nt_ttgABC-5′UTR_ and the original mutants CR, GR, and TR showed comparable tolerance phenotypes (Online Resource Fig. S13–Fig. S15). When the CR and Δ*ttgR* or the GR and Δ*ttgT* strains were complemented with a plasmid for expression of the *ttgR* or the *ttgT* gene, respectively, the mutant strains showed a similar sensitivity towards the monoterpenoids as the wild-type strain. These results confirm that deletion of both the *ttgR* and the *ttgT* gene as well as the deletion of 10 bp in the region between the *ttgR* and *ttgA* open reading frames are causal for the observed monoterpenoid-hypertolerant phenotypes of CR, GR, and TR.

Analysis of the genome sequence data obtained for the GAR mutant revealed a deletion of 41 nucleotides in the *ttgA* gene, coding for a subunit of the TtgABC efflux system. The deletion results in a stop codon after nucleotide 207 of the open reading frame.

In the genome of VR1, a variety of different mutations were identified (Online Resource Table S2). Because none of the identified mutations could be directly linked to an increased monoterpenoid tolerance, a second mutant selected in the presence of verbenone (VR2) was investigated. VR2 showed similar tolerance phenotypes in the tolerance assays compared with VR1 (Fig. 3, Online Resource Fig. S6–Fig. S12) and again over 30 different mutations were identified in its genome (Online Resource Table S2). In both strains, a mutation was present in a gene encoding a DNA repair protein (VR1: *mutS*, VR2: *mutL*). In addition, the genomes of both strains contained a mutation in one of the *nuo* genes (VR1: *nuoB*, VR2: *nuoG*), encoding subunits of NADH-quinone oxidoreductase/NADH dehydrogenase, which is part of the respiratory chain.

As the analysis of VR1 and VR2 genome sequences did not reveal indications for efflux pump alterations, we aimed to test a putative detoxification of verbenone by degradation or conversion by these mutants. However, this hypothesis could be disproven by GC-MS analyses. No conversion products were detectable, and verbenone concentration decreased in the VR cultures over time with the same rate as with WT cells and in the medium control without cells (Online Resource Fig. S16).

Quantitative PCR analysis of mRNA levels of *ttg* genes *ttgR*, *ttgA*, *ttgB*, *ttgT*, and *ttgE* showed reduced expression of TtgR and increased production of TtgABC efflux system in the CR mutant (Fig. 4c). In the TR mutant, transcription of the *ttgABC* efflux pump genes were also significantly enhanced. The opposite was the case with the GAR mutant, which showed a decrease of the TtgABC efflux system mRNA levels. In the GR mutant, the expression of the TtgDEF efflux system was increased. VR1 and VR2 also showed an enhanced mRNA quantity of both efflux pump systems compared with the GS1 wild type.

To measure the efflux activity of the different strains, an efflux pump activity assay was performed according to a protocol by Vidal-Aroca and colleagues (2009). The experiments revealed a reduced resazurin efflux activity for the GAR mutant compared with the wild-type strain (Fig. 4d). While no difference in resazurin efflux activity could be measured between the GR mutant and the wild type, an increased efflux was observed for the CR, TR, and VR1 mutant in the experiments.

### Geranic acid biotransformation performance of monoterpenoid-hypertolerant strains

**Fig. 5:**
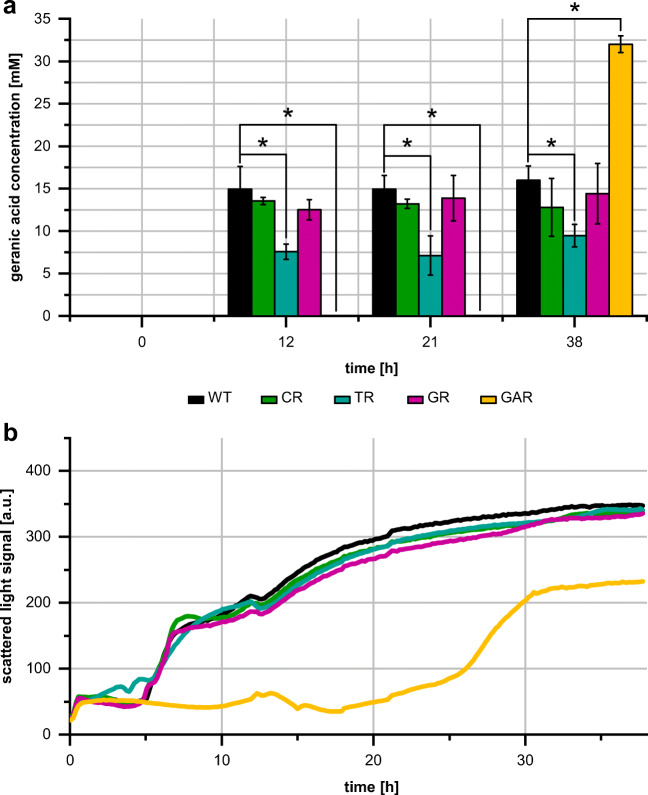
Geranic acid production (**a**) and growth (**b**) of *P. putida* GS1 WT and mutants (CR, TR, GR, GAR) in a geraniol to geranic acid biotransformation. The experiment was conducted in a microbioreactor system over 38 h. To determine geranic acid concentration, samples were taken at time points *t* = 0, 12, 21, and 38 h and analyzed via HPLC. The data points represent the mean values and standard deviations of three biological replicas. The asterisks indicate a significant difference for the mutant strains as compared with wild-type GS1 according to van der Waerden normal score test with Benjamini-Yakutiel *p* value adjustment (*p* < 0.05). Biomass formation was monitored every 10–15 min via scattered light signal intensity (absorbance at 620 nm). The data points represent the mean values of three biological replicas. For variations in growth between the replicas of each strain, see Online Resource Fig. S29

For efficient and robust microbial monoterpenoid de novo production or biotransformation processes, both tolerance of the cells towards educts and products, and productivity should be as high as possible, whereby interactions between these parameters are possible. In order to investigate if the altered monoterpenoid tolerance properties of the mutant strains influence their bioconversion abilities, they were employed for the conversion of geraniol into geranic acid. *P. putida* GS1 has been demonstrated to carry out this biotransformation (Mi et al. 2014). Geraniol (35 mM) was added directly after inoculation of the cultures in the microbioreactor system, and geranic acid concentration was determined at different time points within 38 h (Fig. 5).

In the wild-type culture, a product concentration of 15 mM was reached after 12 h, which increased up to 16 mM until 38 h. Using the TR strain, a geranic acid concentration of 9.5 mM was observed after 38 h. The mutants CR and GR produced geranic acid levels in the range of the wild-type value. Only with the geranic acid mutant GAR, the used geraniol was nearly completely oxidized to 32 mM geranic acid after 38 h. However, the whole conversion took place in the last 14 h of the experiment. This observation was accompanied by the growth curve of GAR. While the wild-type strain and the other mutants started to grow about 5 h after inoculation, cell density of the GAR mutant started to increase after 20 h.

## Discussion

It is long known that some essential oils and their monoterpenoid compounds exhibit antimicrobial properties. However, the specific bacterial mechanisms causing the natural tolerance of, e.g., *Pseudomonas putida* strains towards specific monoterpenoids have hardly been explored. A deeper understanding of the underlying factors and the specificities of the mechanisms is crucial for the development of highly efficient and robust microorganisms producing monoterpenoids de novo or via biotransformation.

In the conducted experiments, the different monoterpenes and monoterpenoids affected the growth behavior of *P. putida* GS1 wild type very differently. None of the tested hydrocarbons and esterified monoterpenoids showed an inhibitory effect on cell growth in the concentrations tested. This indicates that no inhibition of cell functions takes place or that the cells can protect themselves against the substances without affecting growth. Addition of the alcohols, aldehyde, ether, ketone, and acid caused a prolongation of the lag phase, but to different extents. These results are in accordance with the general assumption that the toxicity of a particular substance correlates with its hydrophobicity. The hydrophobicity determines to what extent the compound accumulates in the cytoplasmic membrane. A measure of the hydrophobicity is the logarithm of the octanol-water partition coefficient (log*P*_ow_) of the respective substance, with values between 1 and 4.5. The lower the log*P*_ow_ value, the more toxic the substance is (Inoue and Horikoshi 1989; Sikkema et al. 1994; Ramos et al. 2002; Kabelitz et al. 2003; Dunlop 2011). According to the SciFinder database, the log*P*_ow_ value increases as follows: verbenone < terpinen-4-ol < α**-**terpineol < linalool = 1,8-cineole < geraniol < geranic acid < citral < geranyl formate < α**-**terpinyl acetate < geranyl acetate < γ-terpinene < α**-**terpinene.

Apart from the chemical and physical properties that determine the toxicity of the monoterpenes and monoterpenoids, the observed effects also depend on how effectively *P. putida* GS1 can defend itself against the various substances with its inherent mechanisms. While in the genome of *P. putida* DOT-T1E the genes of three different Ttg efflux systems, TtgABC, TtgDEF and TtgGHI, were found (Segura et al. 2003), the frequently used KT2440 strain contains only the TtgABC system (Nelson et al. 2002). Genome sequencing of the GS1 strain revealed two Ttg efflux systems, TtgABC and TtgDEF, which are very similar to the systems in KT2440 and DOT-T1E (Online Resource Table S3). Some of these systems, such as the TtgABC efflux pump, mediate an intrinsic basal solvent tolerance (Ramos et al. 1998; Rojas et al. 2001). Other mechanisms, such as the expression of the *ttgDEF* operon or an increased TtgABC production, first have to be activated (Mosqueda and Ramos 2000; Rojas et al. 2001; Ramos et al. 2015). This can prolong the lag phase in the presence of toxic compounds, because the cells can only protect themselves effectively against the substances after the genes have been expressed. However, the fact that in the presence of verbenone, α-terpineol and terpinene-4-ol growth is delayed for more than 10 h, is probably not only due to the circumstance that transcription of tolerance mechanisms, such as efflux pumps, first has to be induced. Even with activated efflux pumps, the concentration of the substances in the medium must decrease below a certain value before cell growth is no longer inhibited. Experiments with verbenone showed that the monoterpenoid concentration in the cultures decreases over time (Online Resource Fig. S16) due to evaporation. The concentration reached after about 15 h was ca. 15 mM, which did not cause strong inhibitory effects in previous experiments (Online Resource Fig. S1).

In addition to a prolonged lag phase, the presence of α**-**terpineol, verbenone, and geranic acid impaired growth rate and in the case of geranic acid growth yield. These effects can be explained by an alteration of cellular processes and metabolic fluxes in the cells, e.g., due to increased membrane permeability or the inhibition of certain proteins (Sikkema et al. 1994). Furthermore, the high energy demand of efflux pump activity can cause a reduced growth yield (Ramos et al. 1997; Isken et al. 1999).

The positive effect of some compounds on the maximal biomass yield of *P. putida* GS1 might be explained by a metabolization of the monoterpenoids. As described previously, *P. putida* GS1 is not able to utilize geraniol or geranic acid as sole carbon source (Cantwell et al. 1978; Mi et al. 2014), but can oxidize geraniol into geranic acid, thereby forming NADH and FADH_2_ (Mi et al. 2014) and providing the cells with an additional energy source.

To further elucidate the monoterpenoid tolerance mechanisms in *P. putida* GS1, we isolated and characterized monoterpenoid-hypertolerant mutants. Tolerance assays with selected strains showed different extents of tolerance improvement and different specificities. Genome sequencing, gene expression quantification of *ttg* genes, and efflux activity assays provided further insight into differences between the mutant strains on the cellular level.

In the CR and GR strain, transposon insertion in the *ttgR* or *ttgT* repressor gene, respectively, resulted in a constitutive and increased expression of the corresponding efflux pump proteins TtgABC or TtgDEF (Duque et al. 2001; Terán et al. 2007). Constitutive efflux pump expression can improve solvent tolerance (Fukumori et al. 1998; Duque et al. 2001; Rau et al. 2016) and usually results in a clear reduction of the increased lag phase duration caused by the toxic compound.

The 10-nucleotide deletion in the TR mutant covers the region of the *ttgABC*-5′-UTR directly after the transcription start as well as the − 35 region of the *ttgR* promoter (Terán et al. 2003). The hypertolerance phenotype can be a result of increased *ttgABC* mRNA stability or translation rate. Quantitative PCR experiments showed enhanced transcript levels of the *ttgABC* operon in the TR mutant, which fits to increased efflux activity compared with the wild type. This results in improved solvent tolerance (Fukumori et al. 1998; Duque et al. 2001), as shown in the growth experiments with different monoterpenoids.

In the GAR mutant, the deletion in the *ttgA* gene results in a loss of the TtgABC efflux system accompanied by a reduced resazurin efflux activity. The strain showed an increased sensitivity towards the tested monoterpenoid alcohols, 1,8-cineole, and verbenone, indicating that a functional TtgABC efflux system is not only essential for the tolerance towards toluene and different antibiotics (Ramos et al. 1998; Rojas et al. 2001; Martínez-García and de Lorenzo 2011), but also for monoterpenoid resilience of *P. putida* GS1. The relation between an active efflux and improved monoterpenoid tolerance is in accordance with previous findings. Papadopoulos and colleagues reported that in *Pseudomonas aeruginosa,* the MexAB-OprM efflux system plays a decisive role for the strain’s tolerance towards monoterpenoids such as terpinen-4-ol, 1,8-cineole, and α-terpineol and that the absence of a pump subunit or the entire pump leads to a higher sensitivity towards different monoterpenoids (Papadopoulos et al. 2008). Also for *E. coli* (Shah et al. 2013) and eukaryotic systems (Wang et al. 2013), a causal connection between the presence of efflux pumps and tolerance to monoterpenes and monoterpenoids has been described. However, the correlation between efflux pump expression and monoterpenoid tolerance does not explain the increased tolerance of the GAR strain towards geranic acid, because in this case, loss of an efflux pump component increased the tolerance. A possible explanation is based on the presumption that the geranic acid import rate might be much higher compared with those of the other compounds. If such a continuous uptake is accompanied by a concomitant energy-demanding efflux (Ramos et al. 2002), a futile cycle would have been created. This hypothesis would also explain the clearly reduced growth rate and yield of the wild-type strain in the presence of geranic acid, caused by an energy-consuming futile cycle. The loss of TtgABC activity in the GAR mutant would thus interrupt the cycle and allow a higher growth rate again.

Analysis of the VR mutant genomes revealed more than 30 mutations each, but none of them was located in an efflux pump operon. Nevertheless, further experiments showed increased transcription of TtgABC and TtgDEF efflux pump systems concomitant with an enhanced resazurin efflux activity compared with the wild type. Which mutations lead to an increased expression of the efflux pump genes is unclear. However, the high number of mutations in both strains can be explained by mutations in *mutS* or *mutL*, respectively, which are known to cause high mutation rates (Acharya et al. 2003; Brandt 2006). The fact that both independent strains represent mutator strains strongly indicates that the selection procedure with verbenone required a multigenic trait to bring up these highly tolerant strains. A comparison of the additional genome changes revealed that both strains have a point mutation in a *nuo* gene (VR1: *nuoB*, VR2: *nuoG*). *NuoB* and *nuoG* encode subunits of NADH-ubiquinone oxidoreductase, which is part of the respiratory chain (Weidner et al. 1993; Camacho Carvajal et al. 2002). *NuoB* and *nuoG* were already found to be upregulated in *P. putida* S12 cells during toluene stress (Wijte et al. 2011). The *nuo* gene mutations in VR1 and VR2 could possibly lead to an increased catabolism to provide more ATP for monoterpenoid export or other tolerance mechanisms.

To test the applicability of such hypertolerant strains in biotechnological processes, geraniol to geranic acid biotransformation experiments were conducted. After 38 h, a geranic acid concentration of around 16 mM could be achieved with the wild-type strain, which corresponds to a product yield of 45% and is in accordance with previous studies (Mi et al. 2014). The experiments with the mutants showed that the different tolerance levels of the mutants, caused by differences in efflux activity, had an impact on the conversion of geraniol into geranic acid. The two extremes were the mutants TR and GAR. For the TR mutant, characterized by a high monoterpenoid tolerance and high level of resazurin efflux activity, geraniol conversion ability was significantly impaired. In contrast to this, an almost quantitative conversion of geraniol could be achieved with the GAR mutant (product yield 91%). Our explanation for biotransformation performance reduction of the TR strain is an enhanced export of the biotransformation educt geraniol caused by *ttgABC* overexpression. The GAR mutant lacks this efflux pump and therefore presumably has no or strongly reduced geraniol export activity. This can lead to a strong improvement of the reaction yield, since the educt geraniol is available in the cells for bioconversion. The extraordinarily long lag phase, which was caused by the strong reduction or lack of geraniol efflux activity, can be overcome by separation of growth and production phase in a respective process.

In conclusion, our study demonstrates the highly different toxicity levels of certain monoterpenes and monoterpenoids and reveals the Ttg efflux pumps of *P. putida* GS1 to be mainly responsible for tolerance towards many of the tested compounds. The application example furthermore clearly demonstrates that a fine-tuned tailoring of transport and tolerance properties is essential for the design of efficient and robust biotechnological processes.

## Electronic supplementary material

ESM 1 (PDF 3031 kb)
