# Supplementary material for: Investigation of monoterpenoid resistance mechanisms in Pseudomonas putida and their consequences for biotransformations
Source: Appl Microbiol Biotechnol. 2020 Apr 16;104(12):5519–33. doi: 10.1007/s00253-020-10566-3 (PMC7275096; doi:10.1007/s00253-020-10566-3)
Supplement: Supplementary file 1 — (PDF 3031 kb) [file 253_2020_10566_MOESM1_ESM.pdf]

Journal: Applied Microbiology and Biotechnology

Investigation of monoterpenoid resistance mechanisms in *Pseudomonas putida* and their consequences for biotransformations

Florence Miramella Schempp[1,2], Katharina Elisabeth Hofmann[1], Jia Mi[1], Ferdinand Kirchner[1], Annika Meffert[1], Hendrik Schewe[1], Jens Schrader[1], Markus Buchhaupt[1#]

[1]*DECHEMA-Forschungsinstitut, Industrial Biotechnology, Theodor-Heuss-Allee 25, 60486 Frankfurt am Main, Germany*

[2]*Faculty Biological Sciences, Goethe University Frankfurt, Max-von-Laue-Str. 9, 60438 Frankfurt am Main, Germany*

[#]Address correspondence to Markus Buchhaupt. Phone: +49-69-7564-629, Fax: +49-69-7564-388, e-mail: markus.buchhaupt@dechema.de.

Electronic supplementary material as Online Resource

Table S1. Bacterial strains, plasmids and oligonucleotides used in this study.

Table S2. Identified mutations in the strains TR, GR, GAR, VR1 and VR2.

Table S3. Identified Ttg efflux pump proteins in *P. putida* GS1 and comparison with homologues in *P. putida* strains DOT-T1E, KT2440 and S12.

Table S4. Summary of genome sequencing results and detected mutations.

Fig. S1 – S32. Further results of monoterpenoid tolerance assays.

Supplementary tables

Table S1. Bacterial strains, plasmids and oligonucleotides/PCR primers used in this study.

**Strains**	**Description**	**Reference**
P. putida GS1	alias DSM 12264, wild type *P. putida* isolated from sludge of wastewater treatment system in the Netherlands	DSMZ, Braunschweig, Germany, (Speelmans et al. 1998)
P. putida GS1 CR	*ttgR*::Tn, Tet^R, mutant selected in the presence of 60 mM 1,8-cineole	this work
P. putida GS1 GR	*ttgT*::Tn, Tet^R, mutant selected in the presence of 65 mM geraniol	this work
P. putida GS1 GAR	Δ41 nucleotides of *ttgA*, mutant selected in the presence of 90 mM geranic acid	this work
P. putida GS1 VR1	see Table S2, Tet^R, mutant selected in the presence of 35 mM verbenone	this work
P. putida GS1 VR2	See Table S2, Tet^R, mutant selected in the presence of 35 mM verbenone	this work
P. putida GS1 TR	Δ10 nucleotides of *ttgABC*-5'UTR and -35 region of *ttgR* promoter, Tet^R, mutant selected in the presence of 35 mM α-terpineol	this work
P. putida GS1 Δ*ttgR*	Δ*ttgR*	this work
P. putida GS1 Δ*ttgT*	Δ*ttgT*	this work
P. putida GS1 $\Delta10nt_{ttgABC\text{-}5'UTR}$	Δ10 nucleotides of *ttgABC*-5'UTR and -35 region of *ttgR* promoter	this work
E. coli S17-1 λpir	TpR SmR *recA*, *thi, pro, hsdR*-M+RP4: 2-*Tc:Mu: Km Tn7 λpir*	(Simon et al. 1983)
E. coli DH5α	*fhuA2* Δ(*argF-lacZ*)U169 *phoA glnV44* Φ80 Δ(*lacZ*)M15 *gyrA96 recA1 relA1 endA1 thi-1 hsdR17*	(Hanahan 1985; Grant et al. 1990)
**Plasmids**		
pALMAR-3	plasmid harboring a mariner transposon used for transposon mutagenesis, Tet^R	kindly provided by Jenal group, Uni Basel (Klebensberger et al. 2007)
pMiS4	expression plasmid derived from pBBR1MCS-2; elements of the rhamnose expression system: promoter rhaPBAD and activator genes *rhaR* and *rhaS*; *mob*, pBBR1 *rep*G159S, MCS, Km^R,	(Mi et al. 2016)

pMiS4-eGFP	expression plasmid derived from pBBR1MCS-2; elements of the rhamnose expression system: promoter rhaPBAD and activator genes *rhaR* and *rhaS*; *mob*, pBBR1 *rep*G159S, eGFP, Km^R,	(Mi et al. 2016)
pMiS4-*ttgR*	expression plasmid derived from pBBR1MCS-2; mob, pBBR1 *rep*G159S, *ttgR* with native promoter region, Km^R,	this work
pMiS4-*ttgT*	expression plasmid derived from pBBR1MCS-2; mob, pBBR1 *rep*G159S, *ttgT* with native promoter region, Km^R,	this work
pEMG	*ori*R6K, *lacZα* with two flanking I-SceI sites, Km^R	(Martínez-García and de Lorenzo 2011)
pEMG-Δ*ttgR*	*ori*R6K, *lacZα* with two flanking I-SceI sites, bearing a 1.6 kb insert for deletion *ttgR* gene, Km^R	this work
pEMG-Δ*ttgT*	*ori*R6K, *lacZα* with two flanking I-SceI sites, bearing a 1.6 kb insert for deletion *ttgT* gene, Km^R	this work
pEMG-Δ10 nt-*ttgABC*	*ori*R6K, *lacZα* with two flanking I-SceI sites, bearing a 1.6 kb insert for deletion of 10 nucleotides in *ttgABC*-5’-UTR, Km^R	this work
pSW-2	*ori*RK2, *xylS*, Pm→I-SceI (transcriptional fusion of I-SceI to *Pm*), Gm^R	(Martínez-García and de Lorenzo 2011)
**Oligonucleotides/PCR primers**		**Purpose**
P1	ATCCTTAACAAAGTGGGCAGTACAACCTCATCTGGCC	Cloning in pEMG (pEMG-Δ*ttgR*)
P2	AAGCTTGCATGCCTGCAGGTCGACTCTAGAGGATCCGCCATTGCTCACCAGCGC	
P3	ACGGCCAGTATAGGGATAACAGGGTAATCTGAATTCCGCTGCTGCCGCTCAGCC	
P4	TGAGGTTGTACTGCCCACTTTGTTAAGGATTGTGAGGGAG	
P5	AGTATAGGGATAACAGGGTAATCTGAATTCCTGCGCTTGCCCATTGGTC	Cloning in pEMG (pEMG-Δ*ttgT*)
P6	GCAAAAGTCCTCTAAACCGTTTCCTTCTGATCCAGG	
P7	TCAGAAGGAAACGGTTTAGAGGACTTTTGCTCGGC	
P8	GCATGCCTGCAGGTCGACTCTAGAGGATCCAGACCGCCTATTTATCTGCCTGG	
P9	AGTATAGGGATAACAGGGTAATCTGAATTCTGTCATTT	Cloning in pEMG

	GCGCAGAGCCG	(pEMG-Δ10nt$_{ttgABC\text{-}5'UTR}$)
P10	AATGCTATCCGGTGGTGCTAAGGAATATACTTACATTCATGG	
P11	GTATATTCCTTAGCACCACCGGATAGCATTTTTTCCAG	
P12	GCATGCCTGCAGGTCGACTCTAGAGGATCCGCTTGAACAGGCTGCCGTC	
P13	TTTTCAGGAAATGCGGTGAGCATCACATCAGAGGATCCTCGGGTCGCTG	Cloning of *ttgR* gene in pMiS4 backbone
P14	GGTAGTCAATAAACCGGTAAGCTTGGATCCTCATTTGCGCAGAGCCGG	
P15	TTTTCAGGAAATGCGGTGAGCATCACATCAACCAGATGGCTGCTAGCGG	Cloning of *ttgT* gene in pMiS4 backbone
P16	GGTAGTCAATAAACCGGTAAGCTTGGATCCTCAACCCGCAAACTCCCGAG	
P17	CGAATCGTAACCGTTCGTACGAGAATTCGTACGAGAATCGCTGTCCTCTCCAACGAGCCAAGA	Splinkerette PCR
P18	GATCTCTTGGCTCGTTTTTTTTTGCAAAAA	
P19	CGAATCGTAACCGTTCGTACGAGAA	
P20	CAACCCTTGGCAGAACATATCC	
P21	TCGTACGAGAATCGCTGTCCTCTCC	
P22	GTCCGCCATCTCCAGCAG	
P23	AGGCATTCGTGAAGTCATGG	qPCR, amplification of *rpoD* gene (Franden et al. 2018)
P24	ATGTAACCGCTGAGAACGTC	
P25	GCTCAAGCGCAGCATATC	qPCR, amplification of *ttgR* gene
P26	CCATGTTTGCCTATGTCG	
P27	CAGTTGTATCAGATCGACCCTG	qPCR, amplification of *ttgA* gene
P28	CAGTTGCTTGTAACGTTCGG	
P29	GTATTCCCGTATGACACCACC	qPCR, amplification of *ttgB* gene
P30	GTACATCACCAGGAACACCAG	
P31	GAAGACGATCAAGTCCGGTG	qPCR, amplification of *ttgT* gene
P32	GGATACCGAGTAATGCCCAAG	
P33	GACAAAACCGGCAAGATGAC	qPCR, amplification of *ttgE* gene
P34	AGCCAAACAAGAGGAAGTCG	

Table S2. Identified mutations in the strains TR, GR, GAR, VR1 and VR2. SNE: single nucleotide exchange, nt: nucleotides.

Strain	Type of mutation / Protein effect		Gene or intergenic region / annotated function	
**TR**	Deletion (10 nt, directly after P_{ttgABC} +1)		Intergenic region between *ttgABC* and *ttgR*	
	SNE (transversion)	Substitution	*eryA*	Erythronolide synthase, modules 3 and 4
**GR**	transposon insertion (40 nt downstream of start codon)		*ttgT*	Transcriptional regulator
**GAR**	Deletion (1x T, 2902 nt downstream of start codon)	Frame Shift	*pleC*	Non-motile and phage-resistance protein (PleC)
	Deletion (41 nt, 109 nt downstream of start codon)	Frame Shift	*ttgA*	Efflux system subunit (TtgA)
**VR1**	SNE (transition)	None	*bluB*	5,6-dimethylbenzimidazole synthase
	Deletion (16 nt, 1243 nt downstream of start codon)	Frame Shift	*mutS*	DNA mismatch repair protein (MutS)
	SNE (transition)	Substitution	*ispD*	2-C-methyl-D-erythritol 4-phosphate cytidylyltransferase
	SNE (transition)	None	*cdsA*	Phosphatidate cytidylyltransferase
	SNE (transition)	None	*kgtP_1*	Alpha-ketoglutarate permease
	SNE (transition)	Substitution	*purP_1*	putative adenine permease (PurP)
	SNE (transition)	None	*betB_1*	NAD/NADP-dependent betaine aldehyde dehydrogenase
	SNE (transition)	None	*ybhF_1*	putative ABC transporter ATP-binding protein (YbhF)
	SNE (transition)	Substitution	*tmoS*	Sensor histidine kinase (TmoS)
	SNE (transition)	None	*fccB*	Sulfide dehydrogenase (flavocytochrome c) flavoprotein chain precursor
	SNE (transition)	Substitution	Fatty acid desaturase	
	SNE (transition)	Substitution	*betT_2*	High-affinity choline transport protein
	SNE (transition)	Substitution	*envZ_1*	Osmolarity sensor protein (EnvZ)
	SNE (transition)	Substitution	*mcpS_4*	Methyl-accepting chemotaxis protein (McpS)
	SNE (transition)	Substitution	*dsbD_1*	Thiol:disulfide interchange protein precursor (DsbD)
	SNE (transition)	Substitution	*dppA_4*	Periplasmic dipeptide transport protein precursor
	SNE (transition)		Intergenic region between *mprF* and tRNA-Methionine	
	SNE (transition)	Substitution	Glutamine amidotransferases class-II	
	SNE (transition)	Substitution	*rssA_1*	NTE family protein (RssA)
	SNE (transition)	Substitution	*ydjA*	Putative NAD(P)H nitroreductase (YdjA)
	SNE (transition)	Substitution	*fliC*	B-type flagellin
	SNE (transition)	Substitution	*rutR_2*	HTH-type transcriptional regulator (RutR)
	SNE (transition)	Substitution	*guaD*	Guanine deaminase

	SNE (transition)	Substitution	*lgrB_2*	Linear gramicidin synthase subunit B
	SNE (transition)	Substitution	*adiA*	Biodegradative arginine decarboxylase
	SNE (transition)	Substitution	*nuoB*	NADH-quinone oxidoreductase subunit B
	SNE (transition)	None	*gntZ*	6-phosphogluconate dehydrogenase, NAD(+)-dependent, decarboxylating
	SNE (transition)	Substitution	*alkB_1*	Alkane 1-monooxygenase
	SNE (transition)		Intergenic region between *ssuA2* and *ssuA3*	
	SNE (transition)	Substitution	Hypothetical protein	
	SNE (transition)	None	*atzE*	Biuret hydrolase
	SNE (transition)	Substitution	*puuC_2*	Aldehyde dehydrogenase (PuuC)
	SNE (transition)	Substitution	*mcpS*	Methyl-accepting chemotaxis protein (McpS)
	SNE (transition)	Substitution	*actP_1*	Cation/acetate symporter (ActP)
	SNE (transition)	Substitution	*serB_1*	Phosphoserine phosphatase (SerB_1)
	SNE (transition, 2842 nt downstream of start codon)	Substitution	*pleC*	Non-motile and phage-resistance protein (PleC)
	SNE (transition)	None	*cmpR_2*	HTH-type transcriptional activator (CmpR)
	SNE (transition)	Substitution	*rapA_1*	RNA polymerase-associated protein (RapA)
	SNE (transversion)	Substitution	*mutL*	DNA mismatch repair protein (MutL)
	SNE (transition)	Substitution	*dbpA*	ATP-dependent RNA helicase (DbpA)
	SNE (transition)	Substitution	*gltB*	Glutamate synthase [NADPH] large chain precursor (GltB)
	SNE (transition)		Intergenic region between hypothetical protein and *XpsE* (Type II secretion system protein E)	
	SNE (transversion)	Substitution	*exbB_1*	Biopolymer transport protein (ExbB)
	SNE (transition)		Intergenic region between *alkT* (Rubredoxin-NAD(+) reductase) and *hupB* (DNA-binding protein HU-beta)	
	SNE (transition)	Substitution	*pstC_1*	Phosphate transport system permease protein (PstC)
	SNE (transition)	Substitution	*soxC_2*	Dibenzothiophene desulfurization enzyme C (SoxC)
	SNE (transition)	Substitution	*dinF*	DNA-damage-inducible protein F
	SNE (transversion)		Intergenic region between *yigZ* and *csgA*	
	SNE (transition)	Substitution	*fdx_2*	2Fe-2S ferredoxin
	SNE (transition)	Substitution	*epsE_1*	Type II secretion system protein E
	SNE (transition)	Substitution	hypothetical protein	
	Insertion (tandem repeat – 1x G, 433 nt downstream of start codon)	Frame Shift	*nuoG*	NADH-quinone oxidoreductase subunit G
	SNE (transition)		tRNA-Glycine	
**VR2**	SNE (transition)	Substitution	*proP_5*	Proline/betaine transporter
	SNE (transition)	Substitution	hypothetical protein	
	SNE (transition)	None	*ureC*	Urease subunit alpha
	SNE (transition)	Substitution	*pobB*	Phenoxybenzoate dioxygenase subunit beta
	Deletion (tandem repeat – 1x C, 1035 nt downstream of start	Frame Shift	*bedA*	Benzene 1,2-dioxygenase system ferredoxin-NAD(+) reductase subunit

	codon)			
	SNE (transition)	Substitution	*atoE*	Short-chain fatty acids transporter
	SNE (transition)		Intergenic region between *dmlR* (HTH-type transcriptional regulator) and NADP-dependent 7-alpha-hydroxysteroid dehydrogenase	
	SNE (transition)	Substitution	Acetyltransferase (GNAT) family protein	
	SNE (transition)	None	*ipaH* (putative E3 ubiquitin-protein ligase ipaH4.5)	
	SNE (transition)	Substitution	*hndC*	NADP-reducing hydrogenase subunit (HndC)
	SNE (transition)	Substitution	Hypothetical protein	
	SNE (transition)	None	Hypothetical protein	

Table S3. Identification of Ttg efflux pump proteins in *P. putida* GS1 and comparison with homologues in *P. putida* strains DOT-T1E, KT2440 and S12. Protein identity in %. Pairwise % identity was determined using the software G*eneious* (Biomatters Ltd., Auckland, New Zealand) with *Pairwise alignment* function and standard settings.

**GS1 protein**	**Identity to DOT-T1E**	**Identity to KT2440**	**Identity to S12 Srp proteins**	
TtgR	99.5	99	SrpS	< 10
TtgA	99.5	99.7	SrpA	55.1
TtgB	100	99.8	SrpB	62.9
TtgC	100	99.8	SrpC	56.4
TtgT	100	--	SrpS	62.5
TtgD	99.7	--	SrpA	73.3
TtgE	100	--	SrpB	85.0
TtgF	99.8	--	SrpC	59.6

Table S4. Summary of genome sequencing results and detected mutations. SNE: single nucleotide exchange. [a] Including areas annotated as "hypothetical protein".

**Strain** (*P. putida* GS1)	**Total number of aligned Illumina sequence reads** [Reads]	**Reference genome coverage** [%]	**Average cover per base** [Reads ± standard deviation]	**Total number of mutations**	**Number of SNEs**	**Number of deletions/ insertions**	**Coding regions**	**Non-coding regions** [a]
GR	34,375,723	99.99	213.9 ± 44.5	1	0	1 (transposon insertion)	1	0
TR	30,613,901	99.99	190.5 ± 36.7	2	1	1	1	1
GAR	14,014,168	99.99	154.8 ± 32.6	2	0	2	2	0
VR1	34,341,592	99.99	213.6 ± 39.6	33	32	1	30	3
VR2	48,824,571	99.99	303.9 ± 50.3	32	30	2	24	8

Supplementary figures

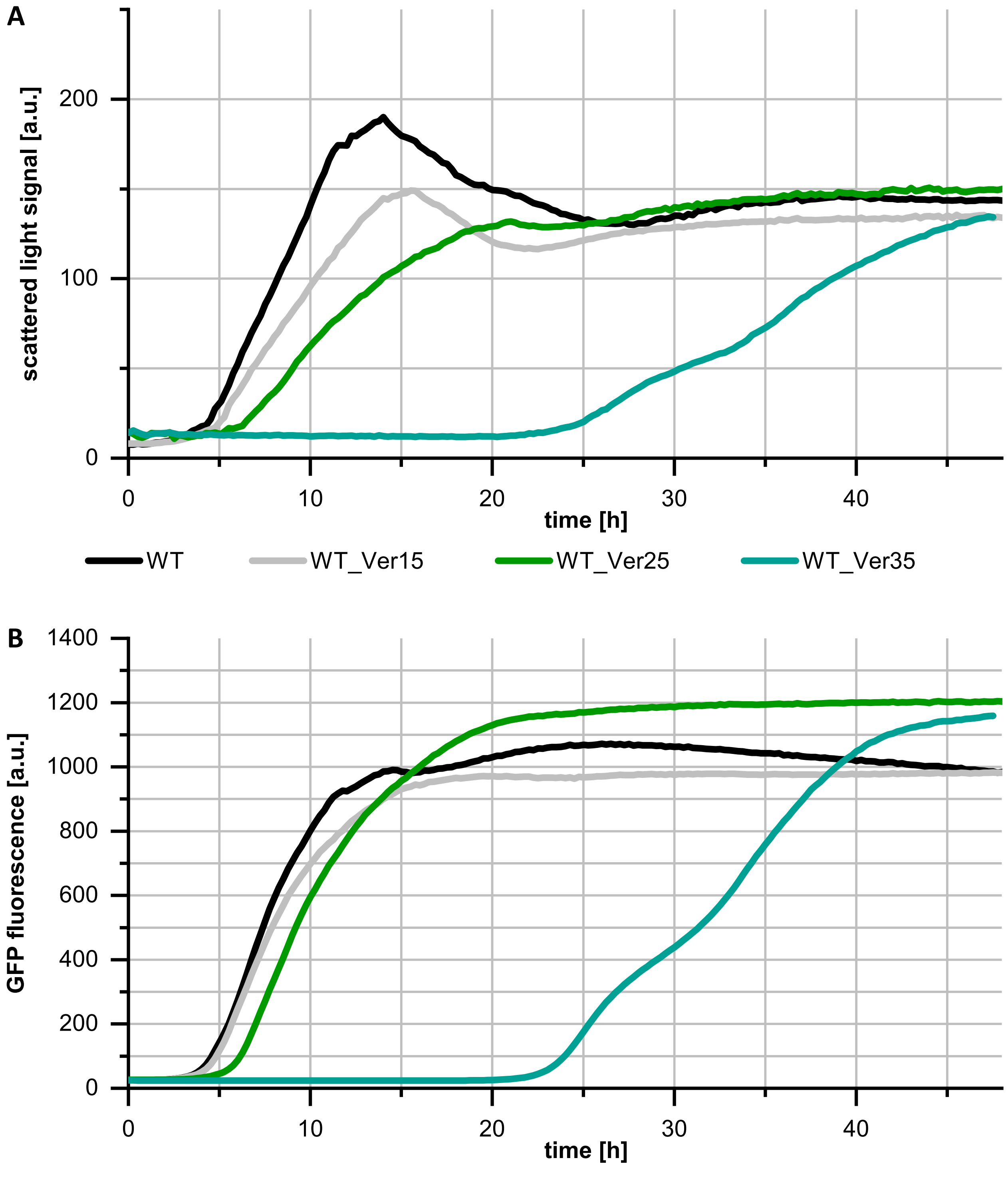

Figure S1. Growth of *P. putida* GS1 WT without and in the presence of different verbenone concentrations (15, 25 and 35 mM). Tolerance assays were conducted in a microbioreactor system over 48 h. Biomass formation was monitored every 10 – 15 minutes via (A) scattered light signal intensity (absorbance at 620 nm) and (B) GFP fluorescence signal intensity (excitation filter: 488 nm; emission filter: 520 nm). The data points represent the mean values of three biological replicas. For variations between the replicas of each strain see Figure S17.

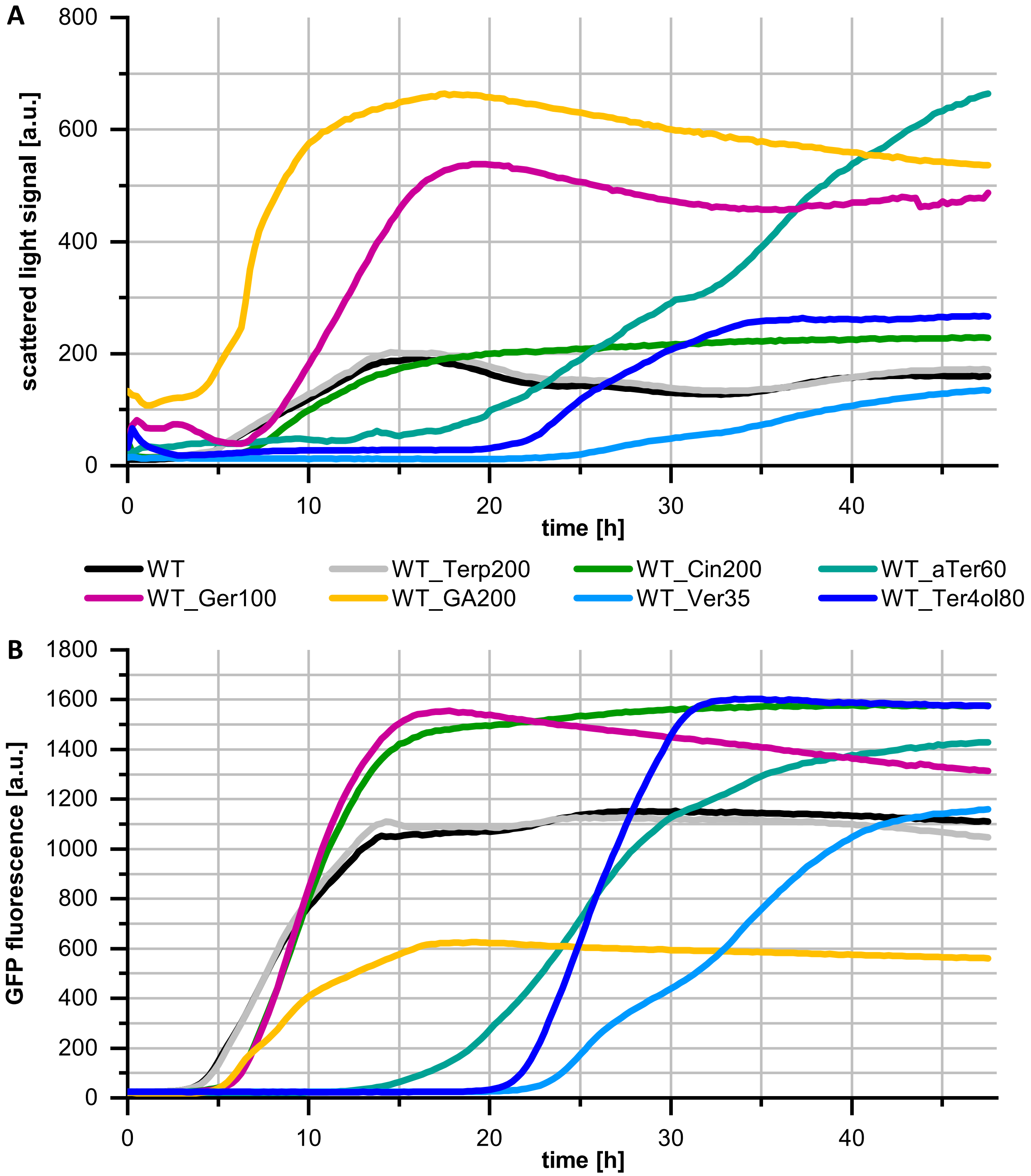

Figure S2. Growth of *P. putida* GS1 (WT) + pMiS4-eGFP without and in the presence of different monoterpenes and monoterpenoids. Terp200: γ-terpinene (200 mM), Cin200: 1,8-cineole (200 mM), aTer60: α-terpineol (60 mM), Ger100: geraniol (100 mM), GA200: geranic acid (200 mM), Ver35: verbenone (35 mM), Ter4ol80: terpinen-4-ol (80 mM). Tolerance assays were conducted in a microbioreactor system over 48 h. Biomass formation was monitored every 10 – 15 minutes via (A) scattered light signal intensity (absorbance at 620 nm) and (B) GFP fluorescence signal intensity (excitation filter: 488 nm; emission filter: 520 nm). The data points represent the mean values of three biological replicas. For variations between the replicas of each strain see Figure S18.

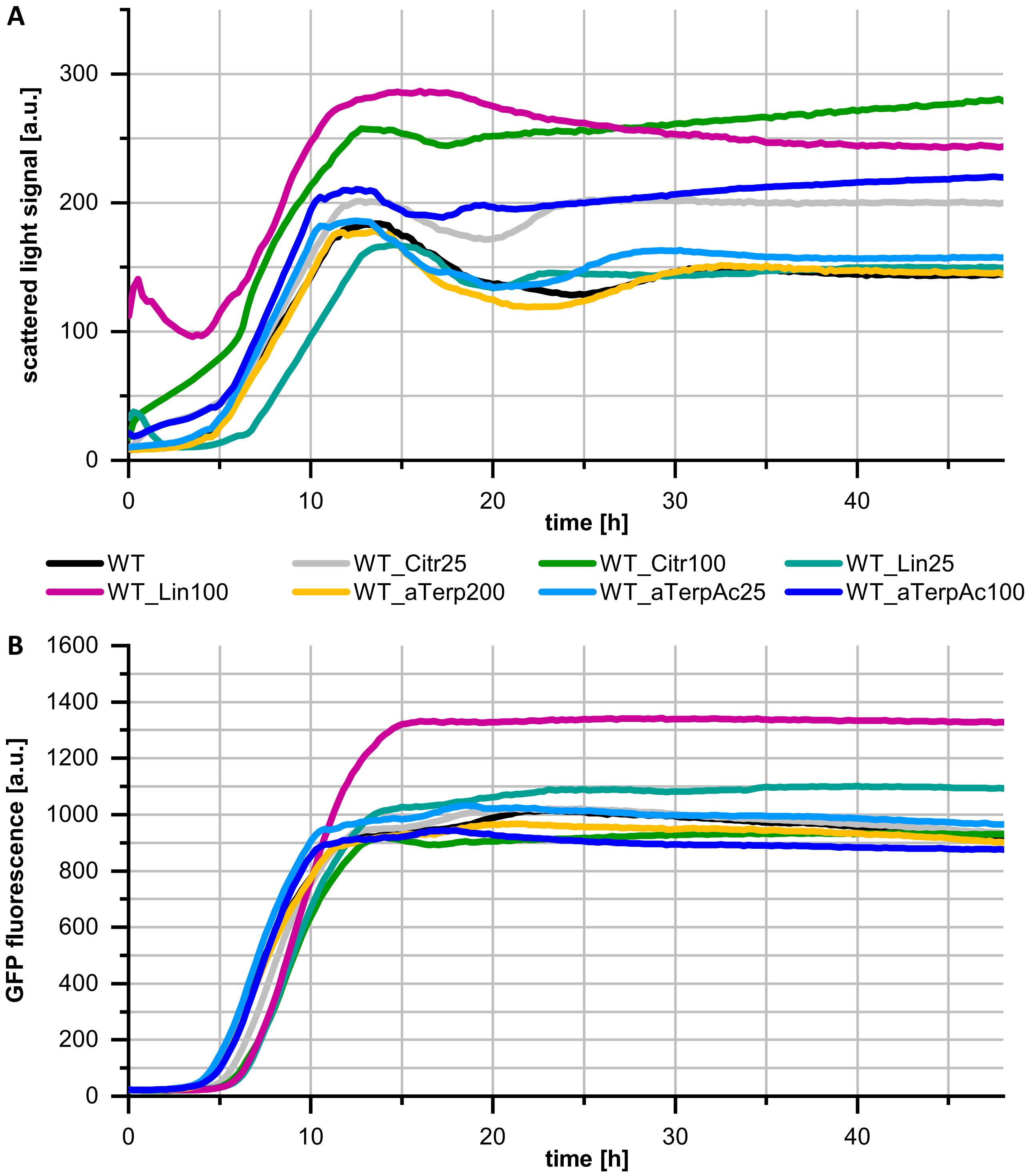

Figure S3. Growth of *P. putida* GS1 (WT) + pMiS4-eGFP without and in the presence of different monoterpenes and monoterpenoids. Citr25/100: citral (25/100 mM), Lin25/100: linalool (25/100 mM), aTerp200: α-terpinene (200 mM), aTerpAc25/100: α-terpinyl acetate (25/100 mM). Tolerance assays were conducted in a microbioreactor system over 48 h. Biomass formation was monitored every 10 – 15 minutes via (A) scattered light signal intensity (absorbance at 620 nm) and (B) GFP fluorescence signal intensity (excitation filter: 488 nm; emission filter: 520 nm). The data points represent the mean values of two or three biological replicas. For variations between the replicas of each strain see Figure S19.

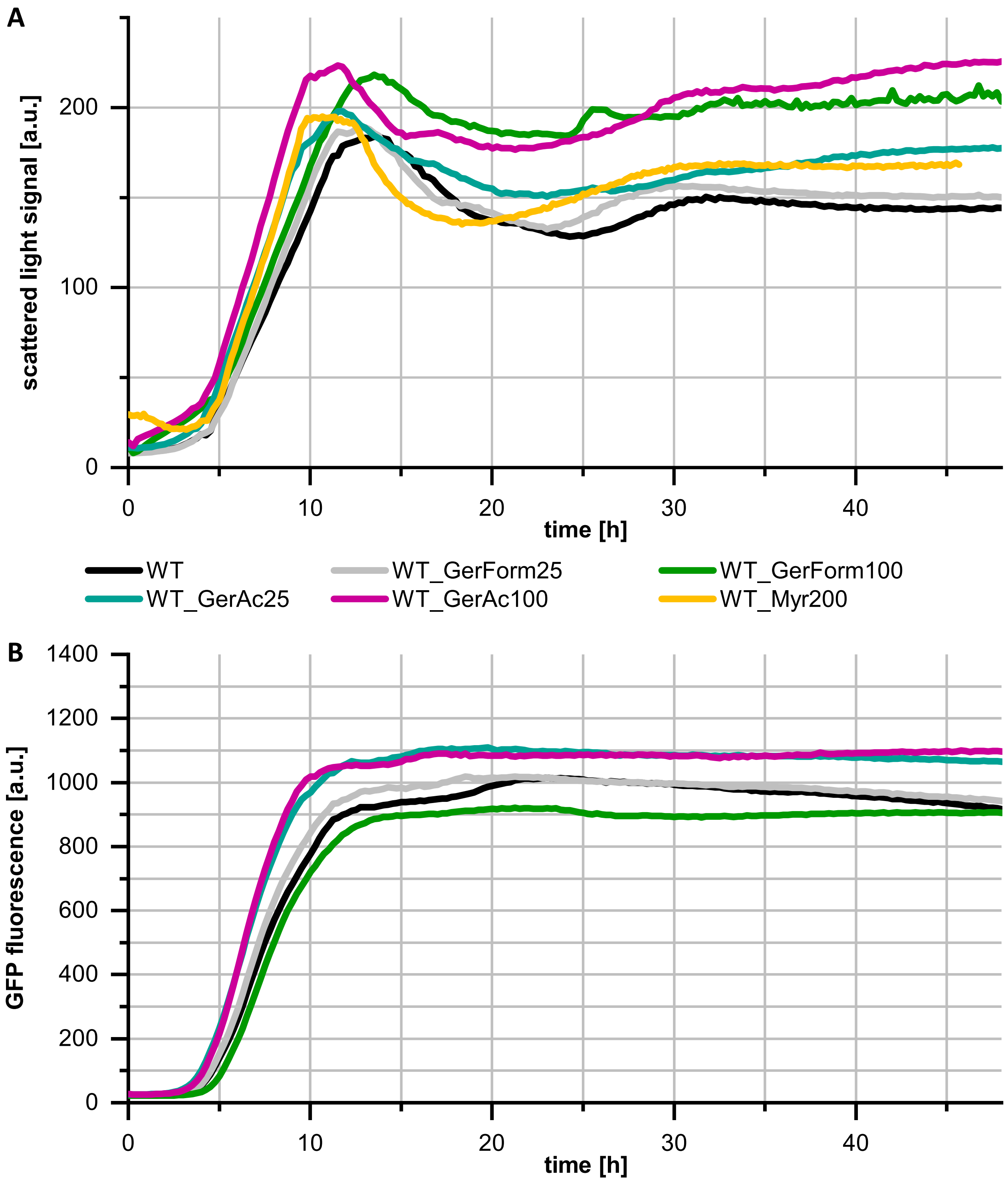

Figure S4. Growth of *P. putida* GS1 (WT) + pMiS4-eGFP without and in the presence of different monoterpenes and monoterpenoids. GerForm25/100: geranyl formate (25/100 mM), GerAc25/100: geranyl acetate (25/100 mM), Myr200: Myrcene (200 mM). Tolerance assays were conducted in a microbioreactor system over 48 h. Biomass formation was monitored every 10 – 15 minutes via (A) scattered light signal intensity (absorbance at 620 nm) and (B) GFP fluorescence signal intensity (excitation filter: 488 nm; emission filter: 520 nm). The data points represent the mean values of two or three biological replicas. For variations between the replicas of each strain see Figure S20.

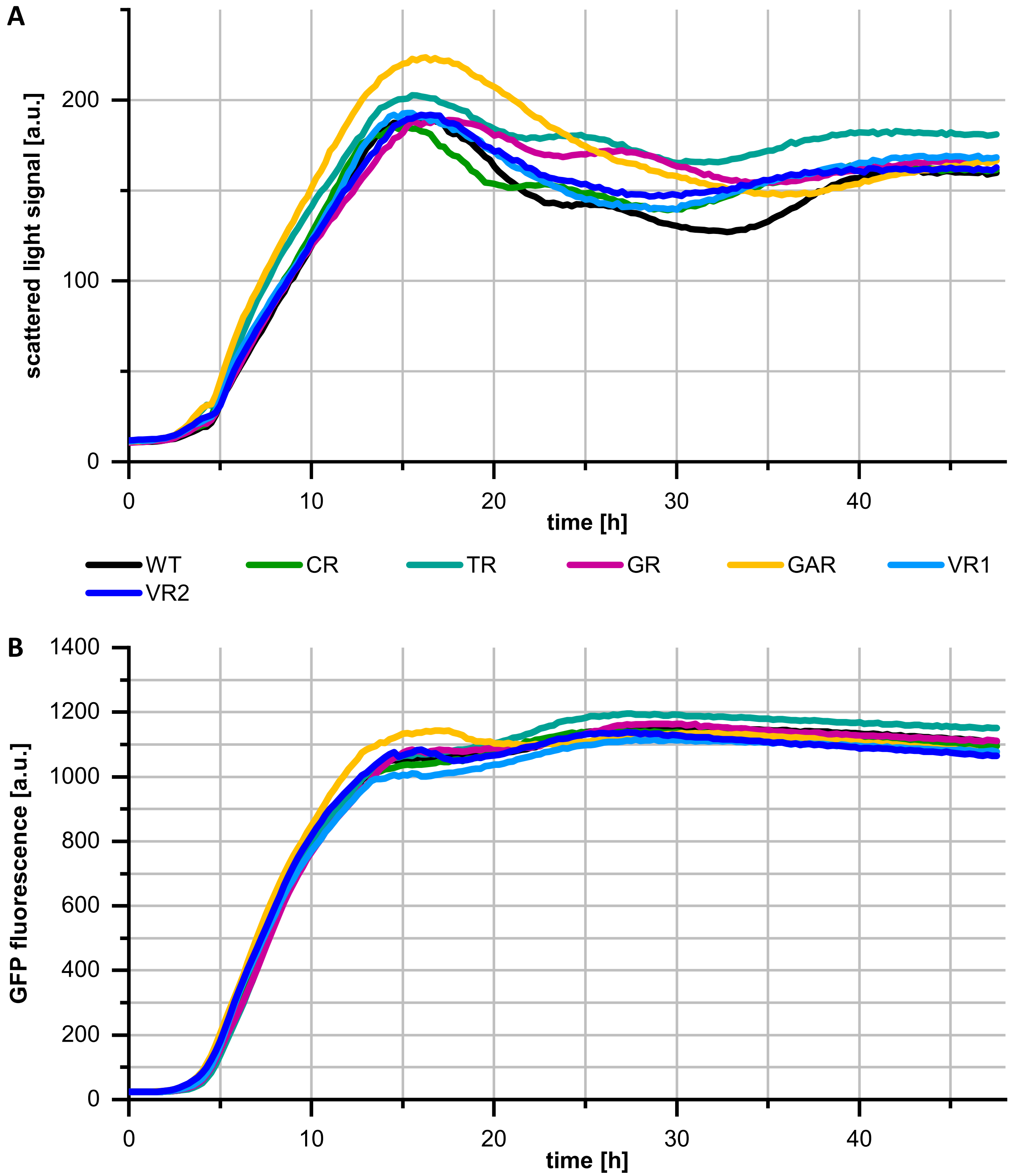

Figure S5. Growth of *P. putida* GS1 WT and mutants + pMiS4-eGFP without monoterpene or monoterpenoid. Tolerance assays were conducted in a microbioreactor system over 48 h. Biomass formation was monitored every 10 - 15 minutes via (A) scattered light signal intensity (absorbance at 620 nm) and (B) GFP fluorescence signal intensity (excitation filter: 488 nm; emission filter: 520 nm). The data points represent the mean values of three biological replicas. For variations between the replicas of each strain see Figure S21.

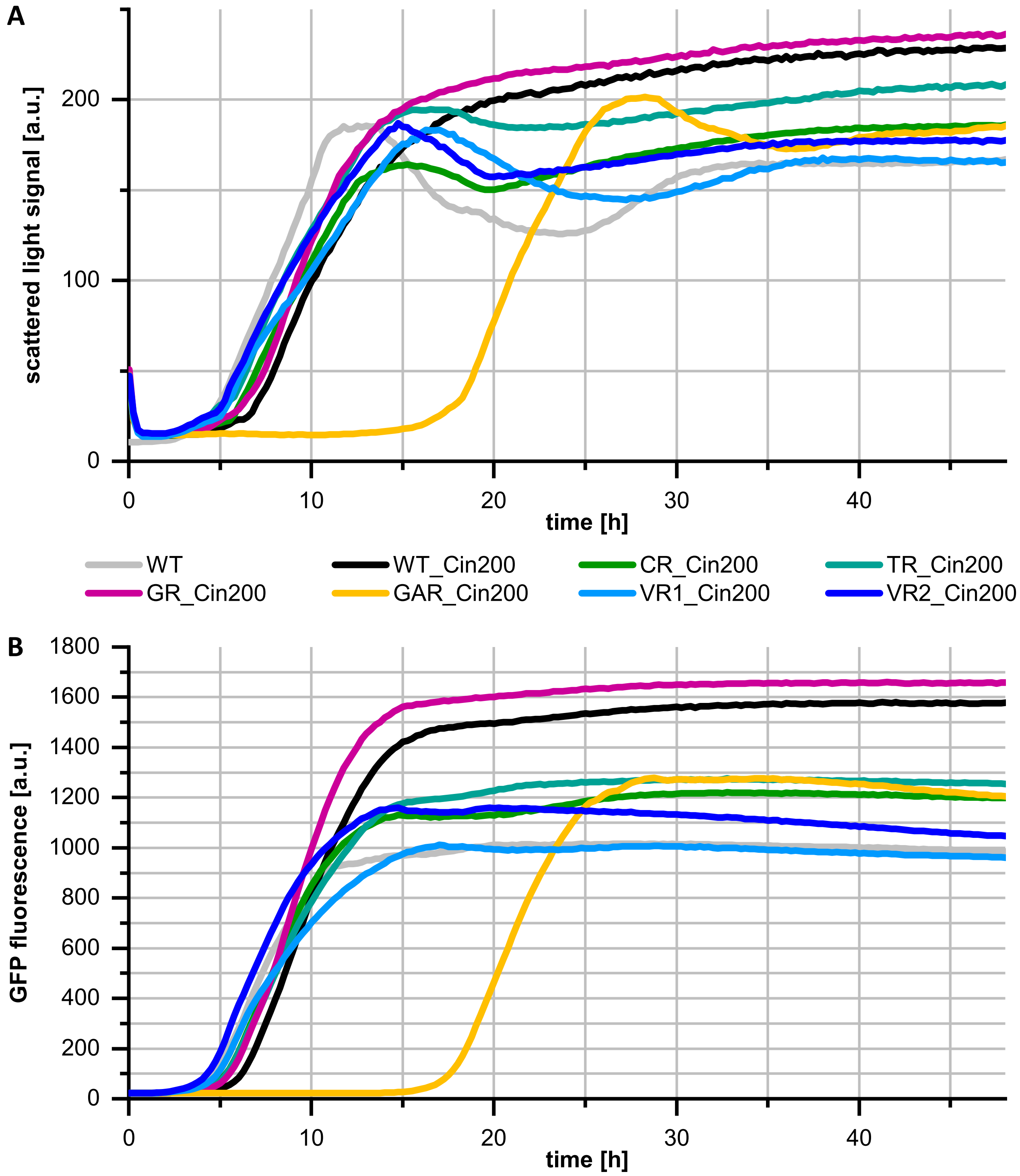

Figure S6. Growth of *P. putida* GS1 WT and mutants + pMiS4-eGFP without and in the presence of 200 mM 1,8-cineole. Tolerance assays were conducted in a microbioreactor system over 48 h. Biomass formation was monitored every 10 - 15 minutes via (A) scattered light signal intensity (absorbance at 620 nm) and (B) GFP fluorescence signal intensity (excitation filter: 488 nm; emission filter: 520 nm). The data points represent the mean values of three biological replicas. For variations between the replicas of each strain see Figure S22.

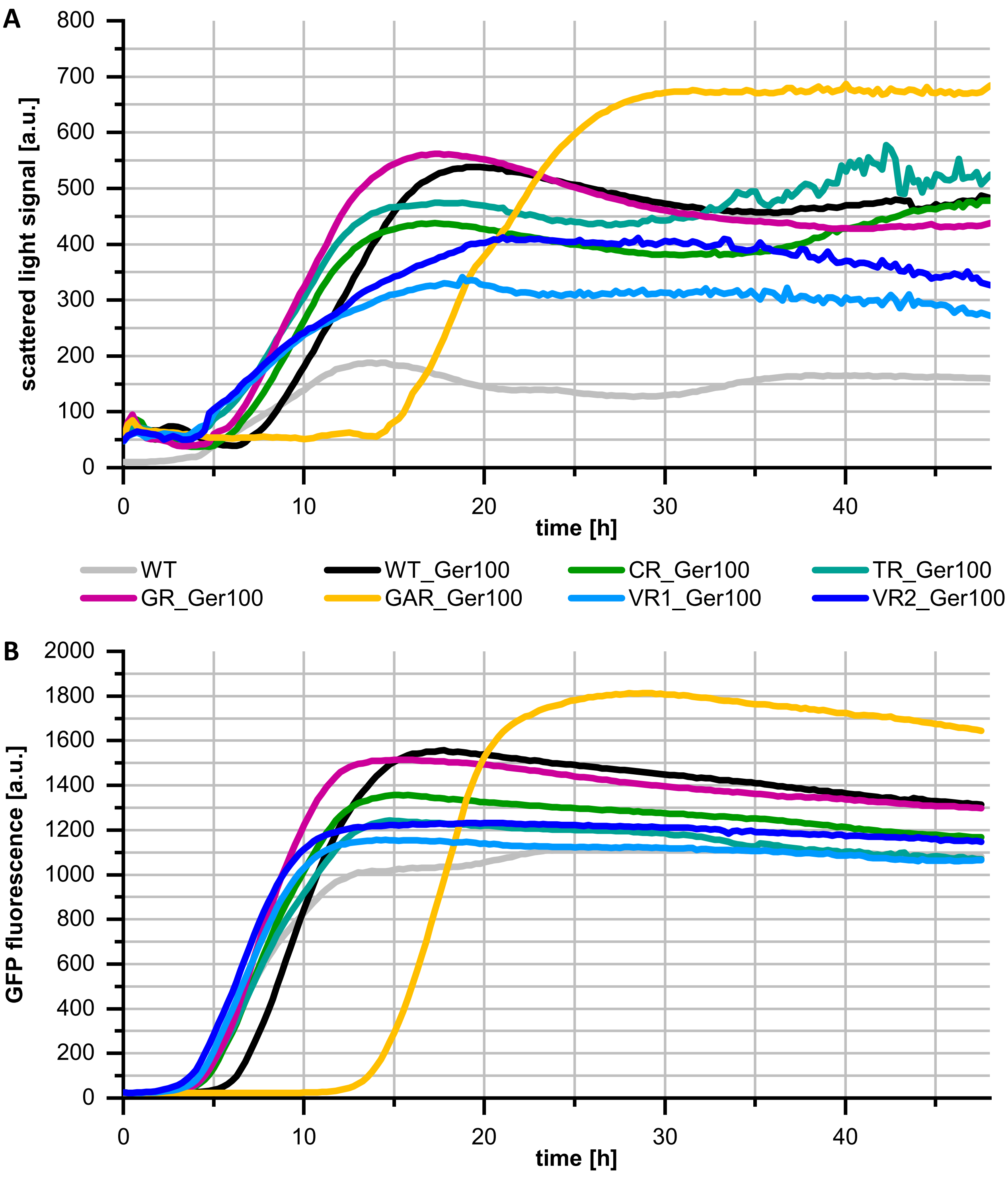

Figure S7. Growth of *P. putida* GS1 WT and mutants + pMiS4-eGFP without and in the presence of 100 mM geraniol. Tolerance assays were conducted in a microbioreactor system over 48 h. Biomass formation was monitored every 10 - 15 minutes via (A) scattered light signal intensity (absorbance at 620 nm) and (B) GFP fluorescence signal intensity (excitation filter: 488 nm; emission filter: 520 nm). The data points represent the mean values of three biological replicas. For variations between the replicas of each strain see Figure S23.

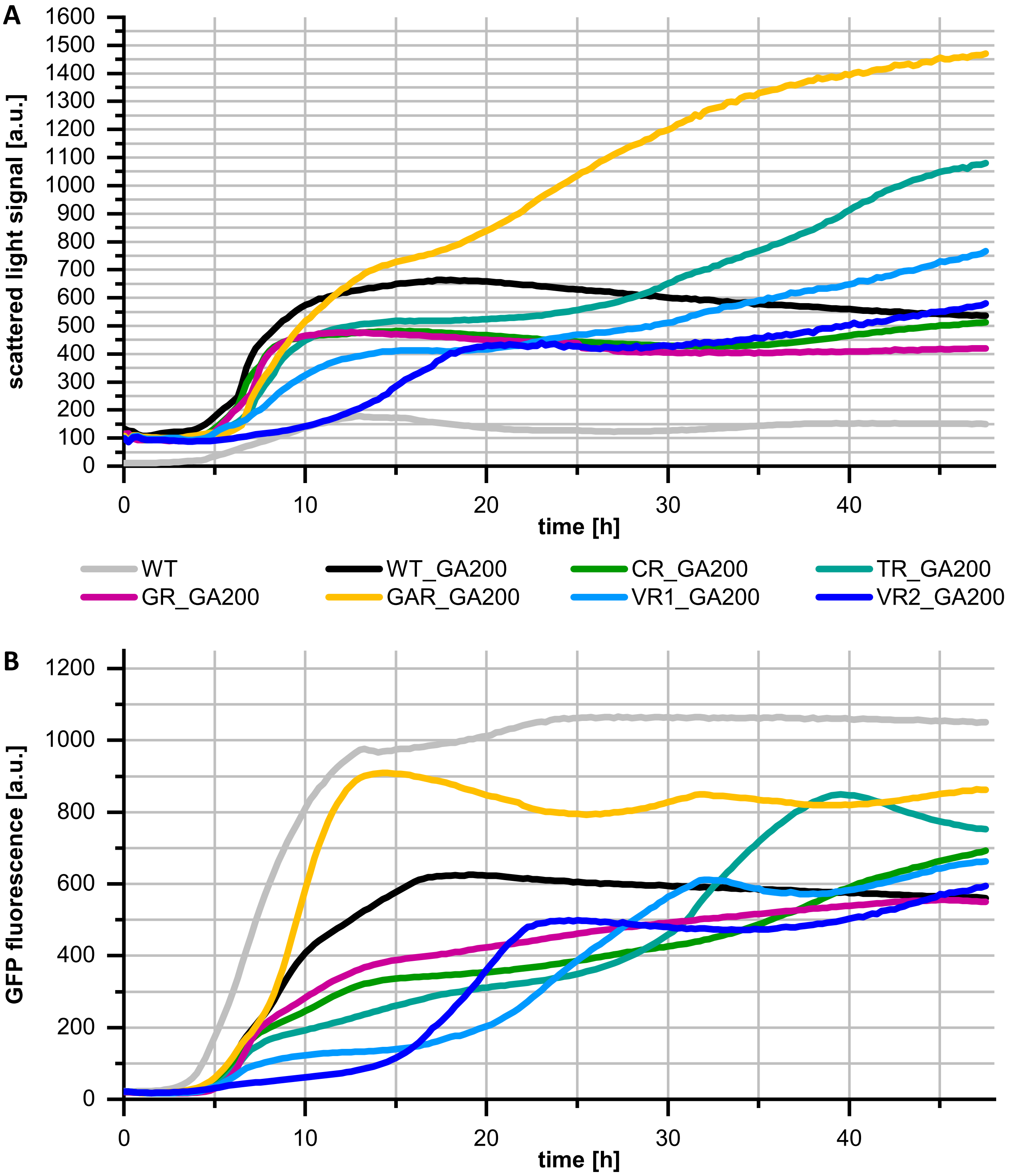

Figure S8. Growth of *P. putida* GS1 WT and mutants + pMiS4-eGFP without and in the presence of 200 mM geranic acid. Tolerance assays were conducted in a microbioreactor system over 48 h. Biomass formation was monitored every 10 - 15 minutes via (A) scattered light signal intensity (absorbance at 620 nm) and (B) GFP fluorescence signal intensity (excitation filter: 488 nm; emission filter: 520 nm). The data points represent the mean values of three biological replicas. For variations between the replicas of each strain see Figure S24.

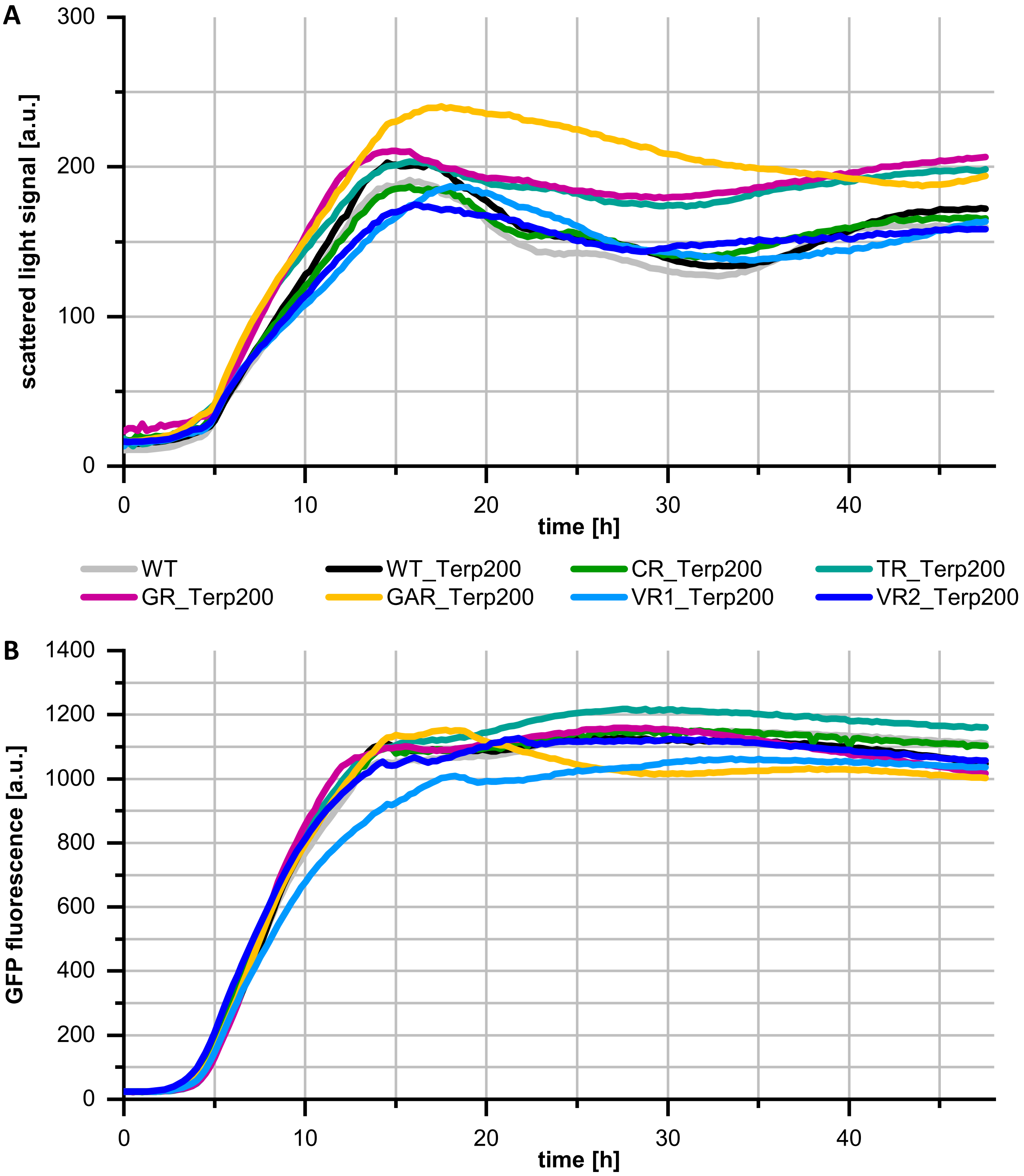

Figure S9. Growth of *P. putida* GS1 WT and mutants + pMiS4-eGFP without and in the presence of 200 mM γ-terpinene. Tolerance assays were conducted in a microbioreactor system over 48 h. Biomass formation was monitored every 10 - 15 minutes via (A) scattered light signal intensity (absorbance at 620 nm) and (B) GFP fluorescence signal intensity (excitation filter: 488 nm; emission filter: 520 nm). The data points represent the mean values of three biological replicas. For variations between the replicas of each strain see Figure S25.

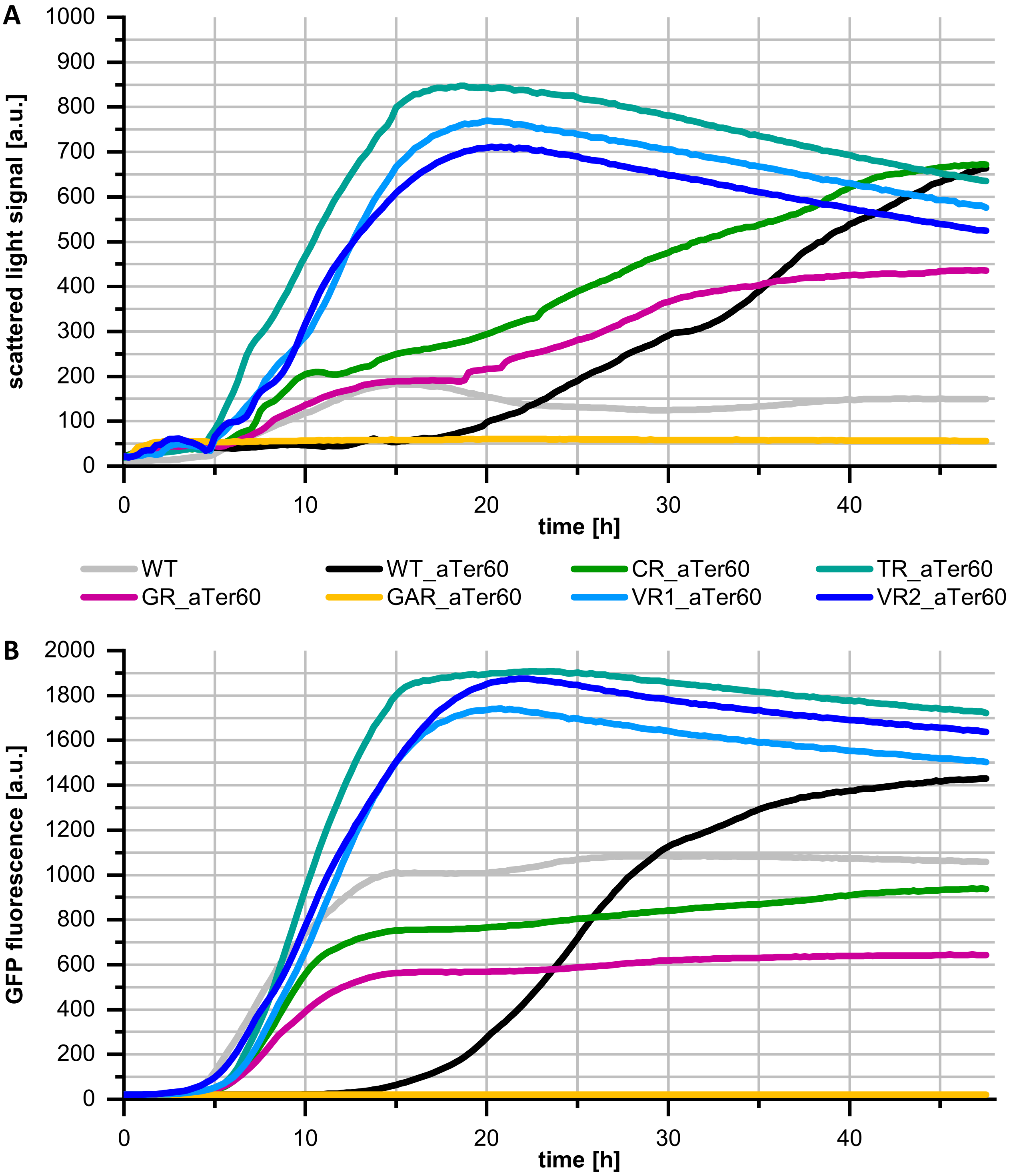

Figure S10. Growth of *P. putida* GS1 WT and mutants + pMiS4-eGFP without and in the presence of 60 mM α-terpineol. Tolerance assays were conducted in a microbioreactor system over 48 h. Biomass formation was monitored every 10 - 15 minutes via (A) scattered light signal intensity (absorbance at 620 nm) and (B) GFP fluorescence signal intensity (excitation filter: 488 nm; emission filter: 520 nm). The data points represent the mean values of two (VR1) or three biological replicas. For variations between the replicas of each strain see Figure S26.

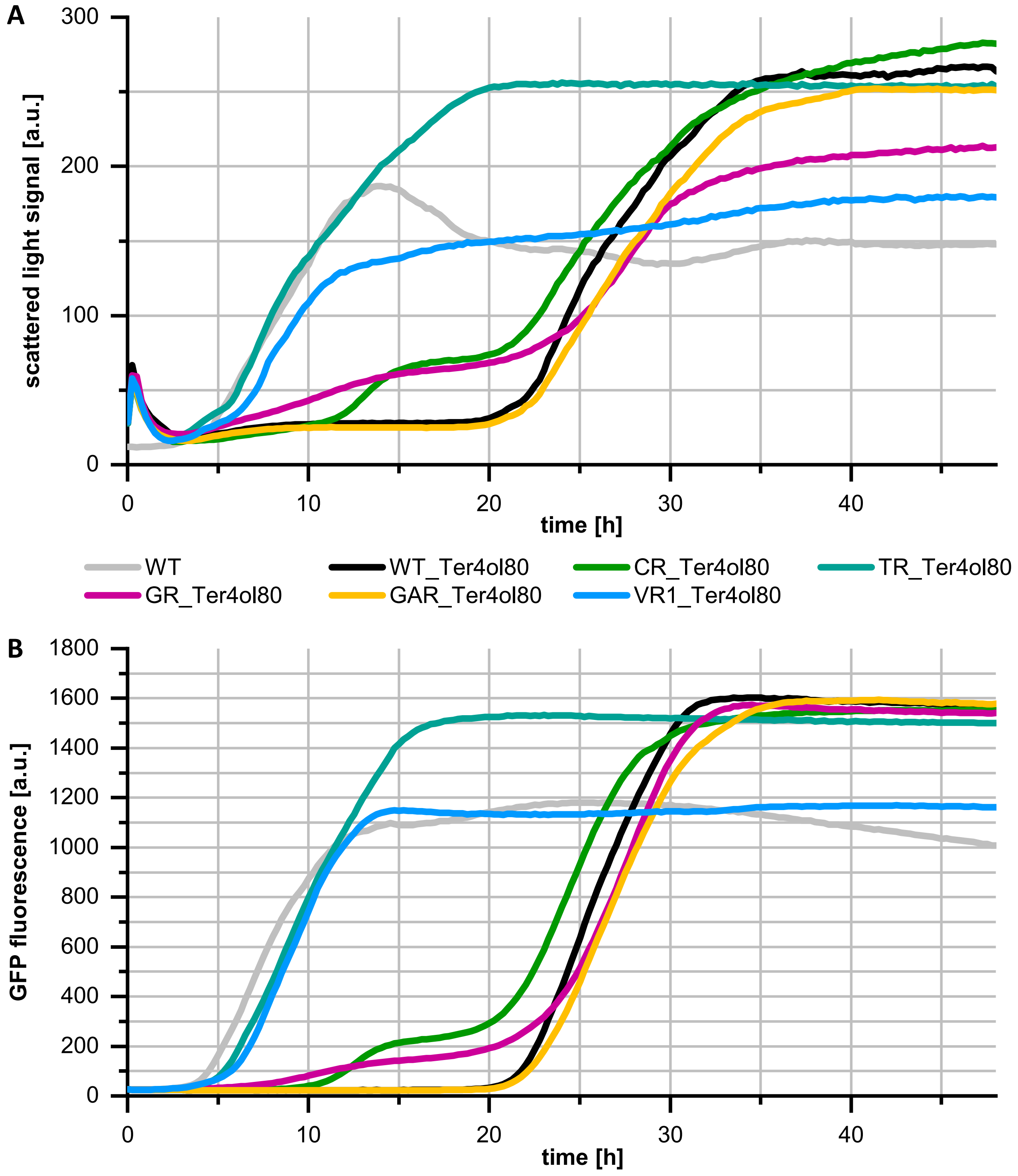

Figure S11. Growth of *P. putida* GS1 WT and mutants + pMiS4e-GFP without and in the presence of 80 mM terpinen-4-ol. Tolerance assays were conducted in a microbioreactor system over 48 h. Biomass formation was monitored every 10 - 15 minutes via (A) scattered light signal intensity (absorbance at 620 nm) and (B) GFP fluorescence signal intensity (excitation filter: 488 nm; emission filter: 520 nm). The data points represent the mean values of two or three biological replicas. For variations between the replicas of each strain see Figure S27.

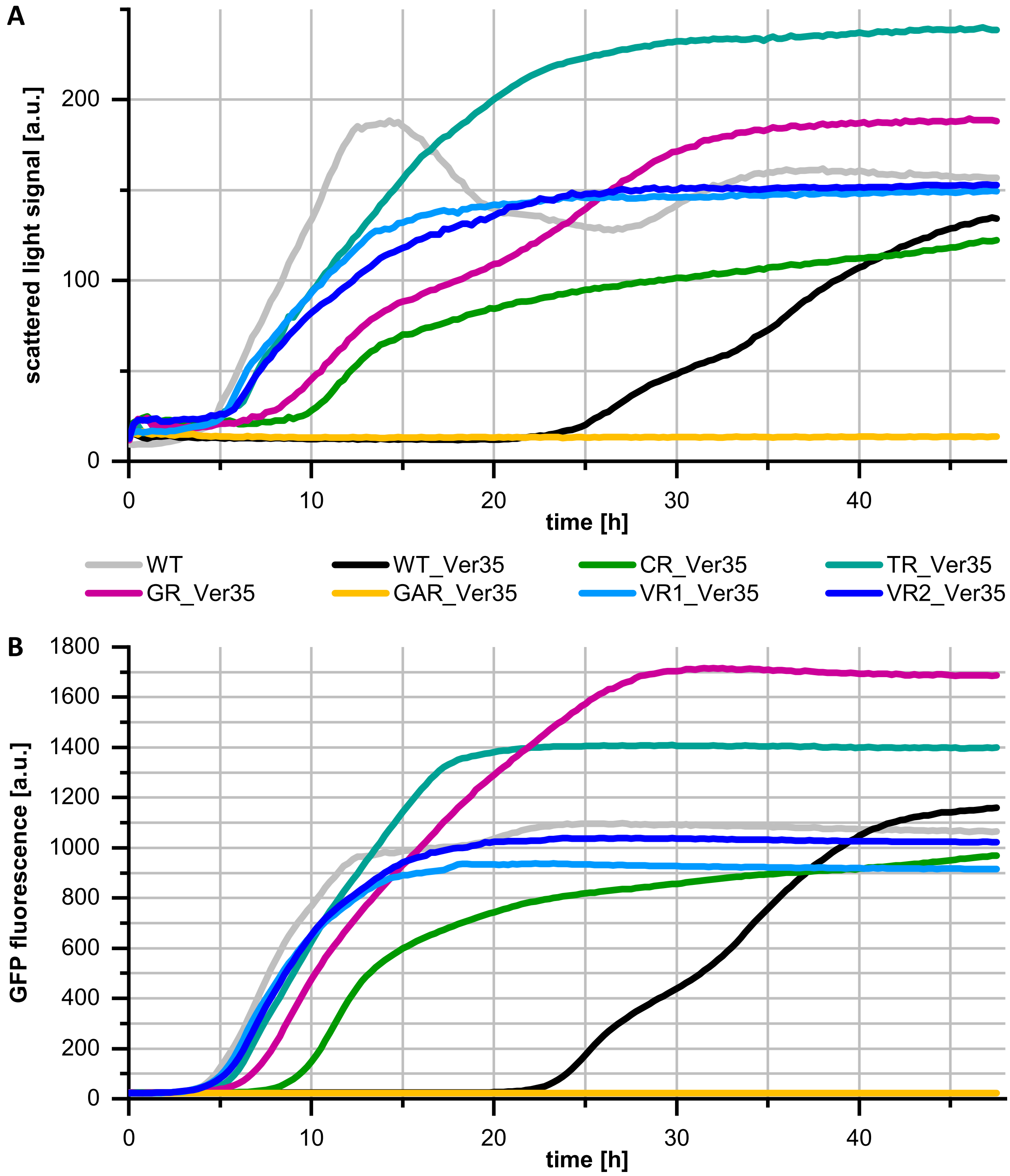

Figure S12. Growth of *P. putida* GS1 WT and mutants + pMiS4-eGFP without and in the presence of 35 mM verbenone. Tolerance assays were conducted in a microbioreactor system over 48 h. Biomass formation was monitored every 10 - 15 minutes via (A) scattered light signal intensity (absorbance at 620 nm) and (B) GFP fluorescence signal intensity (excitation filter: 488 nm; emission filter: 520 nm). The data points represent the mean values of two or three biological replicas. For variations between the replicas of each strain see Figure S28.

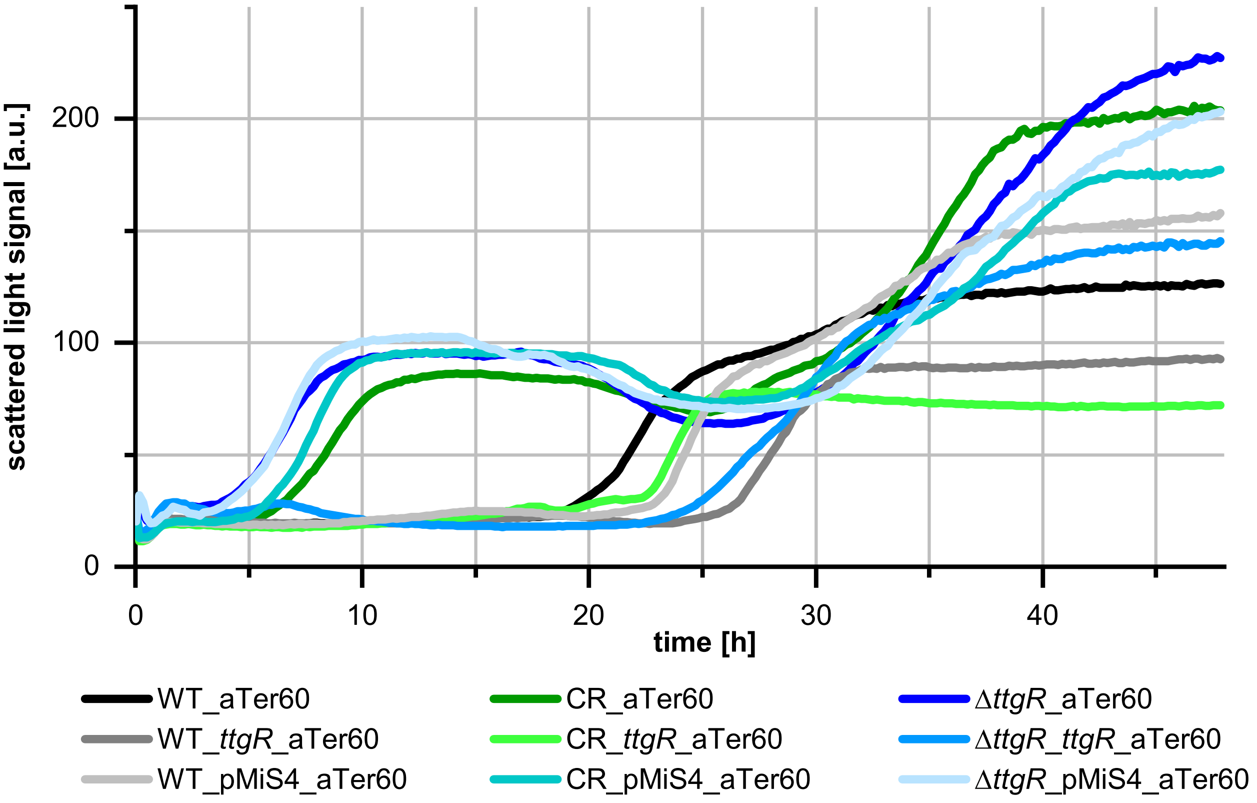

Figure S13. Verification of *ttgR* involvement in α-terpineol-hypertolerance phenotype by deletion and complementation. Growth curves of cultures with 60 mM α-terpineol are shown. _pMiS4_ = strains contain the pMiS4 empty plasmid, _*ttgR*_ = strains contain the pMiS4-*ttgR* vector. Tolerance assays were conducted in a microbioreactor system over 48 h. Biomass formation was monitored every 10 - 15 minutes via (A) scattered light signal intensity (absorbance at 620 nm) and (B) GFP fluorescence signal intensity (excitation filter: 488 nm; emission filter: 520 nm). The data points represent the mean values of three biological replicas. For variations between the replicas of each strain see Figure S30.

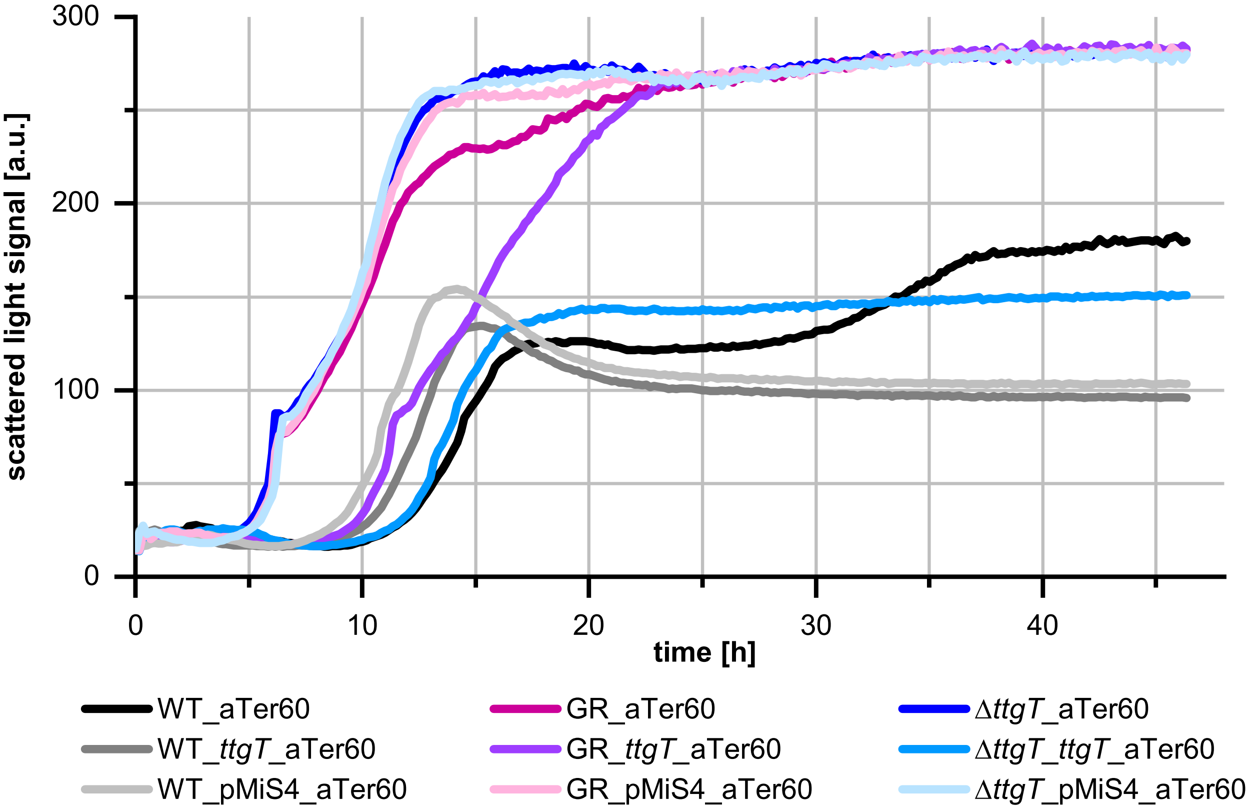

Figure S14. Verification of *ttgT* involvement in α-terpineol-hypertolerance phenotype by deletion and complementation. Growth curves of cultures with 60 mM α-terpineol are shown. _pMiS4_ = strains contain the pMiS4 empty plasmid, _*ttgT*_ = strains contain the pMiS4-*ttgT* vector. Tolerance assays were conducted in a microbioreactor system over 48 h. Biomass formation was monitored every 10 - 15 minutes via (A) scattered light signal intensity (absorbance at 620 nm) and (B) GFP fluorescence signal intensity (excitation filter: 488 nm; emission filter: 520 nm). The data points represent the mean values of three biological replicas. For variations between the replicas of each strain see Figure S31.

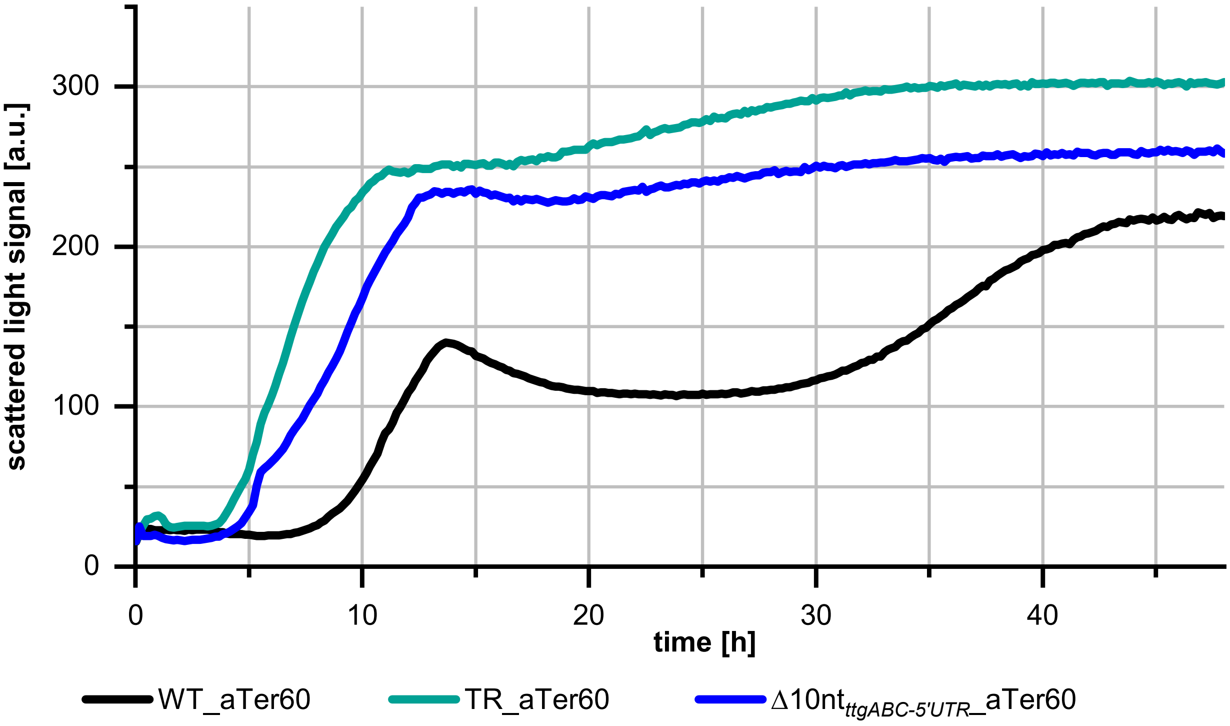

Figure S15. Verification of Δ10nt$_{ttgABC\text{-}5'UTR}$ involvement in α-terpineol-hypertolerance phenotype by deletion. Growth curves of cultures with 60 mM α-terpineol are shown. Tolerance assays were conducted in a microbioreactor system over 48 h. Biomass formation was monitored every 10 - 15 minutes via (A) scattered light signal intensity (absorbance at 620 nm) and (B) GFP fluorescence signal intensity (excitation filter: 488 nm; emission filter: 520 nm). The data points represent the mean values of three biological replicas. For variations between the replicas of each strain see Figure S32.

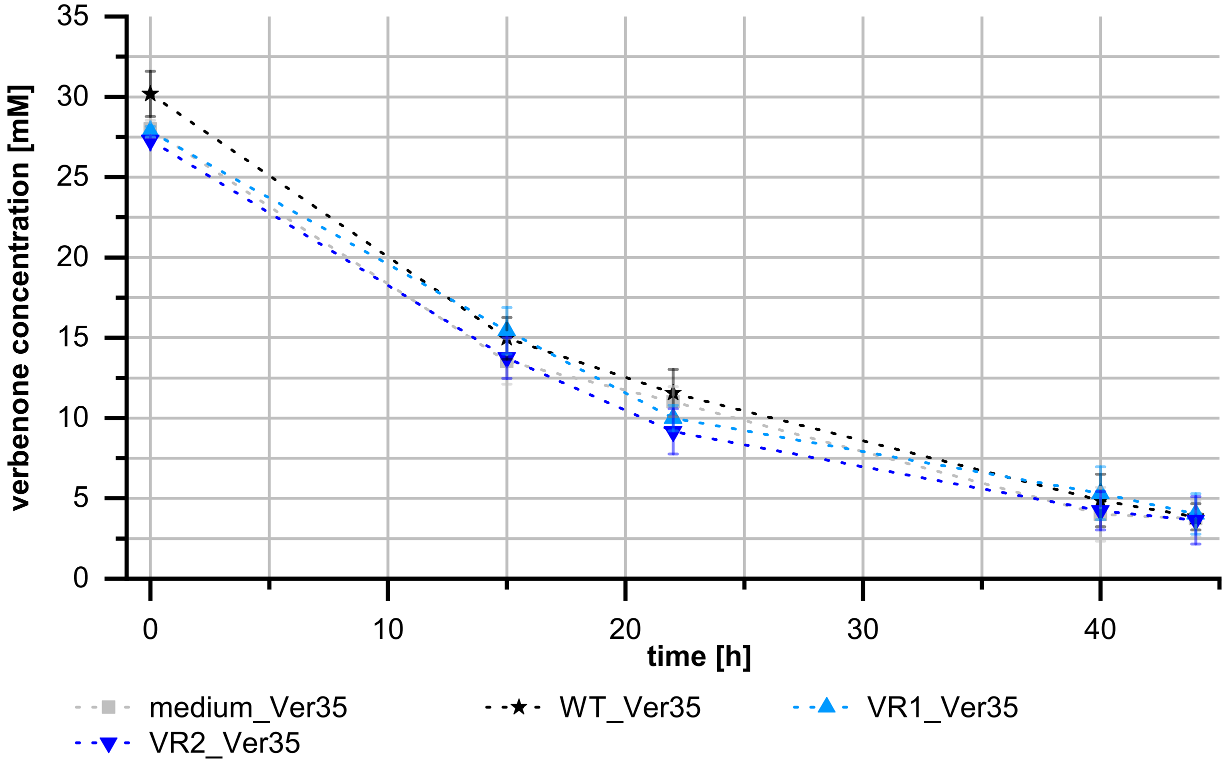

Figure S16. Determination of verbenone concentration in culture medium (WT, VR1, VR2) or in culture free medium (medium) over time. *P.* putida strains were grown in a microbioreactor system over 44 h with initial addition of 35 mM verbenone. To determine verbenone concentration, samples were taken at time points t = 0, 15, 22, 40, 44 h and analyzed via GC-MS. The data points represent the mean values and standard deviations of three biological replicas.

Supplementary note 1

The growth curve figures Fig. 2, Fig. 3 and Fig. 5 as well as Figure S1 - Figure S15 show mean values from cultivations of three biological replicates. To show the variations within the experiments, Figure S17 - Figure S32 present the corresponding individual growth curves.

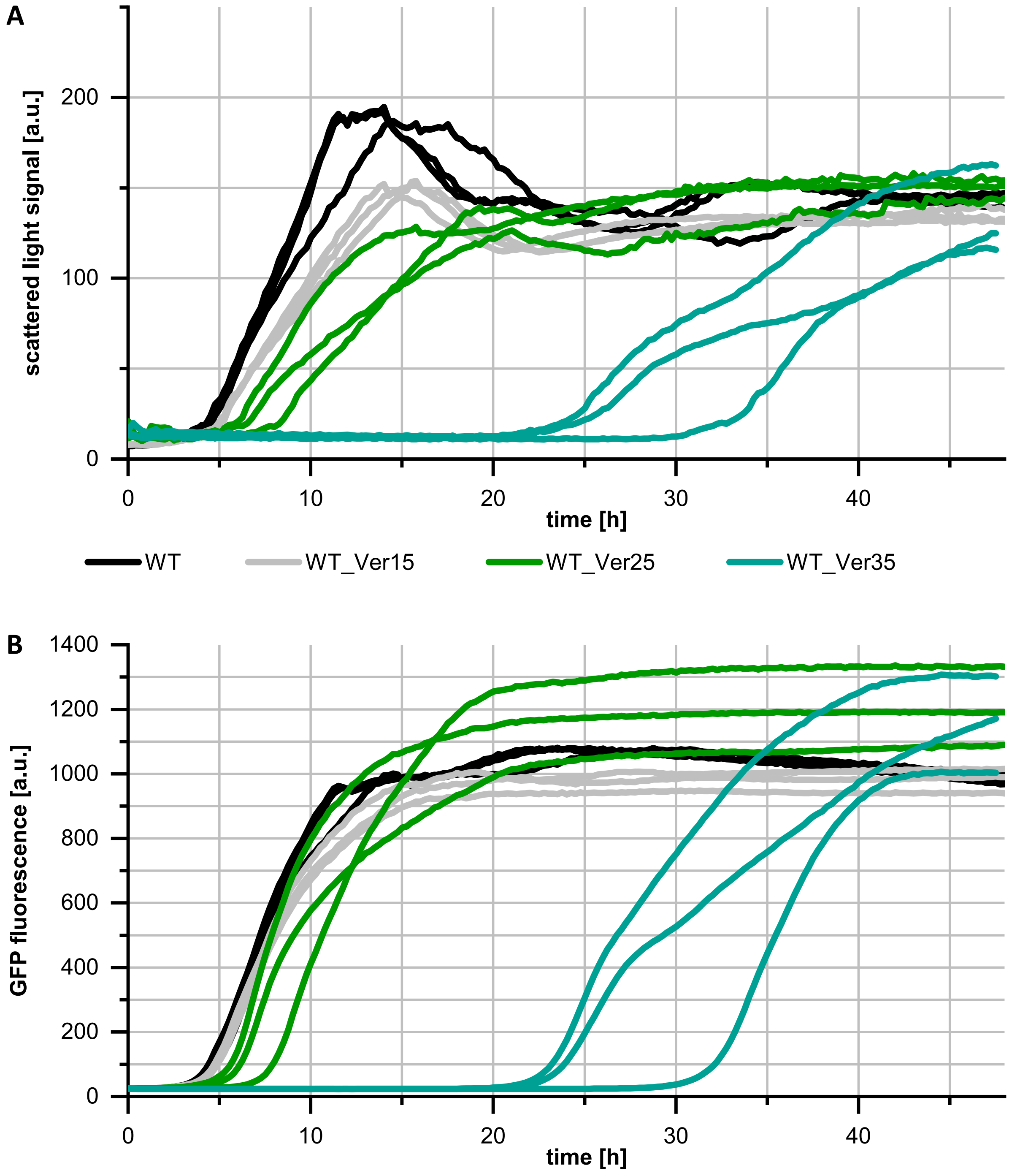

Figure S17. Growth of different *P. putida* GS1 WT cultures without and in the presence of different verbenone concentrations (15, 25 and 35 mM). Tolerance assays were conducted in a microbioreactor system over 48 h. Biomass formation was monitored every 10 - 15 minutes via (A) scattered light signal intensity (absorbance at 620 nm) and (B) GFP fluorescence signal intensity (excitation filter: 488 nm; emission filter: 520 nm).

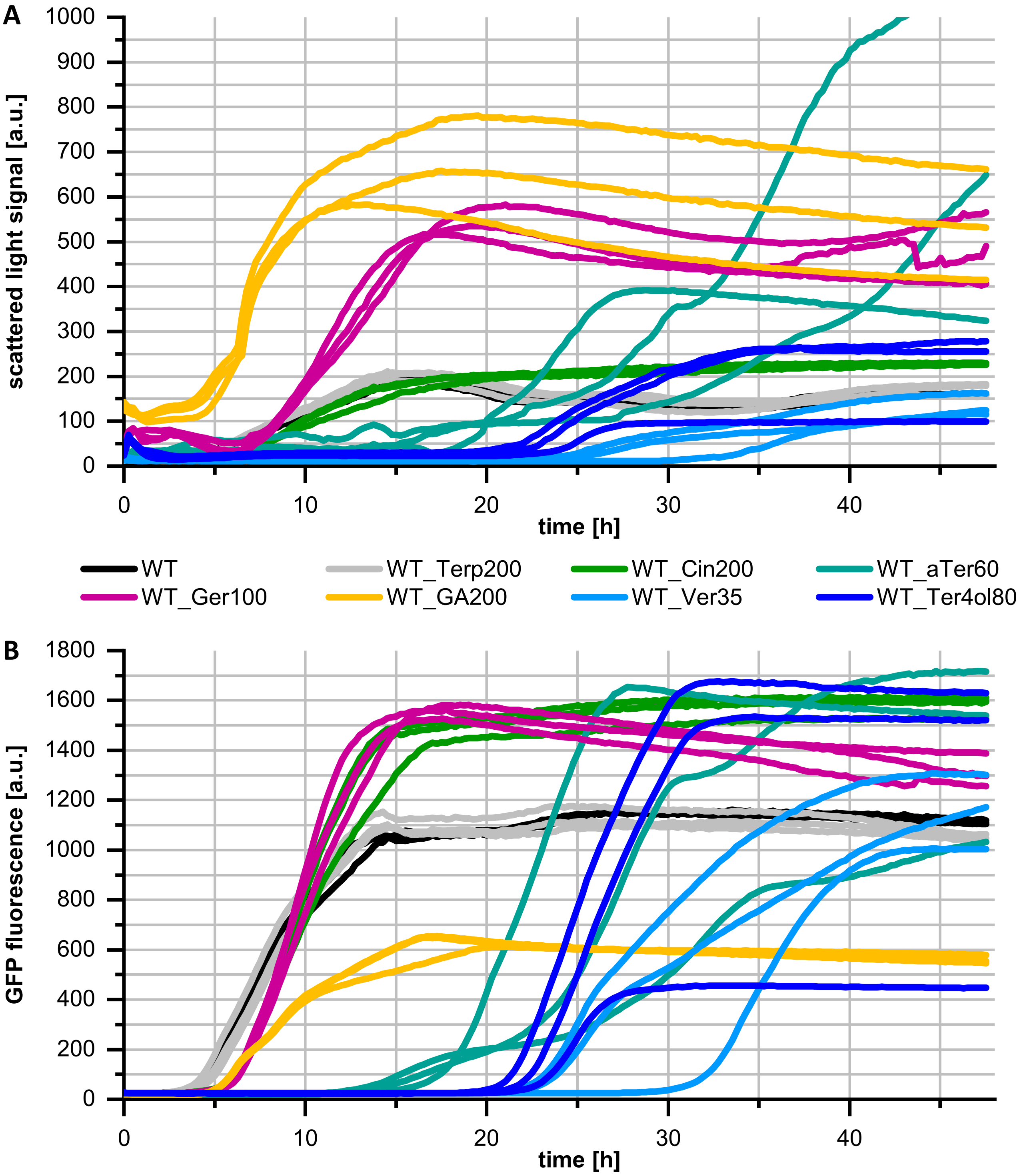

Figure S18. Growth of three different *P. putida* GS1 (WT) + pMiS4-eGFP cultures without and in the presence of different monoterpenes and monoterpenoids. Terp200: γ-terpinene (200 mM), Cin200: 1,8-cineole (200 mM), aTer60: α-terpineol (60 mM), Ger100: geraniol (100 mM), GA200: geranic acid (200 mM), Ver35: verbenone (35 mM), Ter4ol80: terpinen-4-ol (80 mM). Tolerance assays were conducted in a microbioreactor system over 48 h. Biomass formation was monitored every 10 - 15 minutes via (A) scattered light signal intensity (absorbance at 620 nm) and (B) GFP fluorescence signal intensity (excitation filter: 488 nm; emission filter: 520 nm).

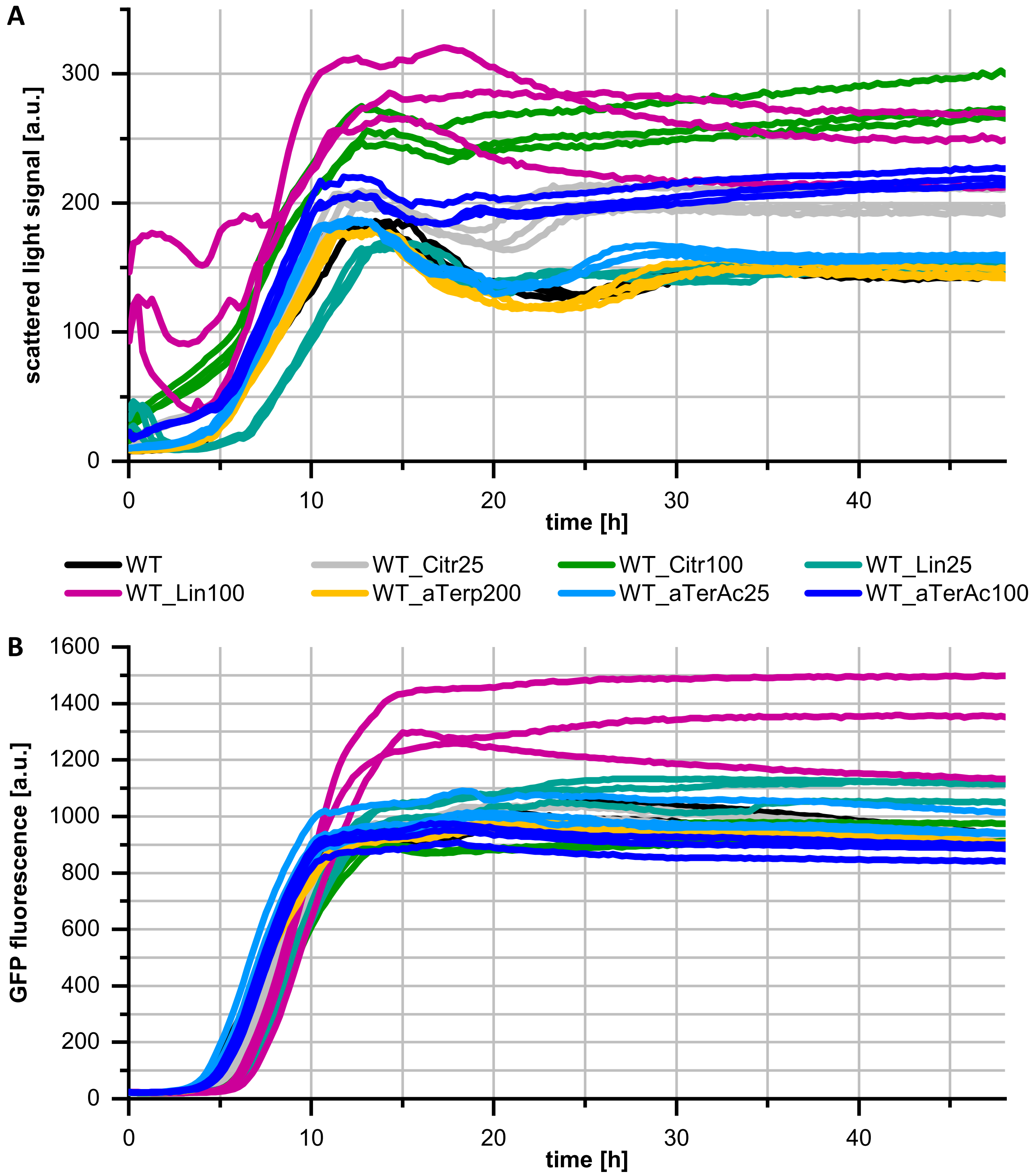

Figure S19. Growth of three different *P. putida* GS1 (WT) + pMiS4-eGFP cultures without and in the presence of different monoterpenes and monoterpenoids. Citr25/100: citral (25/100 mM), Lin25/100: linalool (25/100 mM), aTerp200: α-terpinene (200 mM), aTerpAc25/100: α-terpinyl acetate (25/100 mM). Tolerance assays were conducted in a microbioreactor system over 48 h. Biomass formation was monitored every 10 - 15 minutes via (A) scattered light signal intensity (absorbance at 620 nm) and (B) GFP fluorescence signal intensity (excitation filter: 488 nm; emission filter: 520 nm).

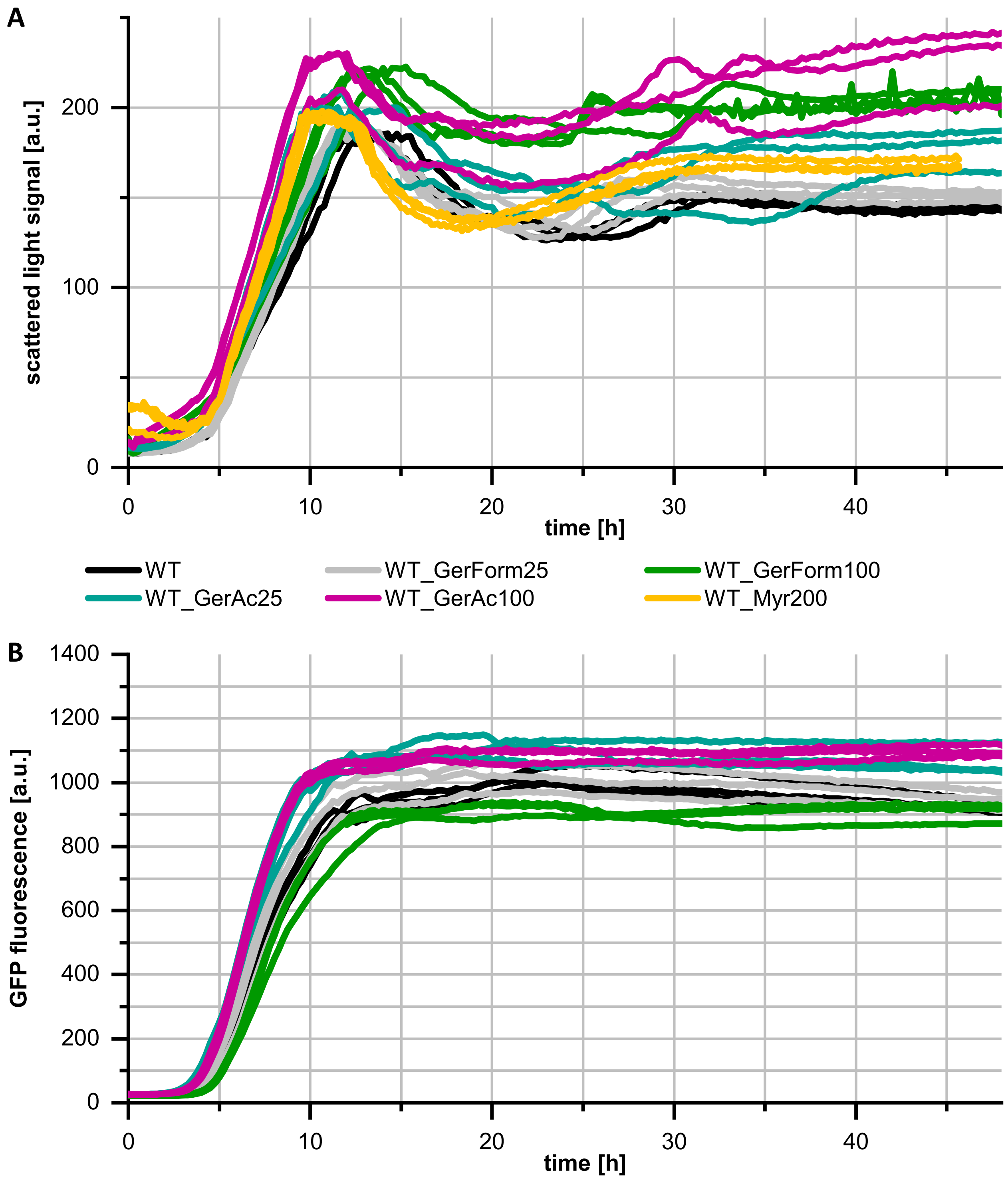

Figure S20. Growth of three different *P. putida* GS1 (WT) + pMiS4-eGFP cultures without and in the presence of different monoterpenes and monoterpenoids. GerForm25/100: geranyl formate (25/100 mM), GerAc25/100: geranyl acetate (25/100 mM), Myr200: Myrcene (200 mM). Tolerance assays were conducted in a microbioreactor system over 48 h. Biomass formation was monitored every 10 - 15 minutes via (A) scattered light signal intensity (absorbance at 620 nm) and (B) GFP fluorescence signal intensity (excitation filter: 488 nm; emission filter: 520 nm).

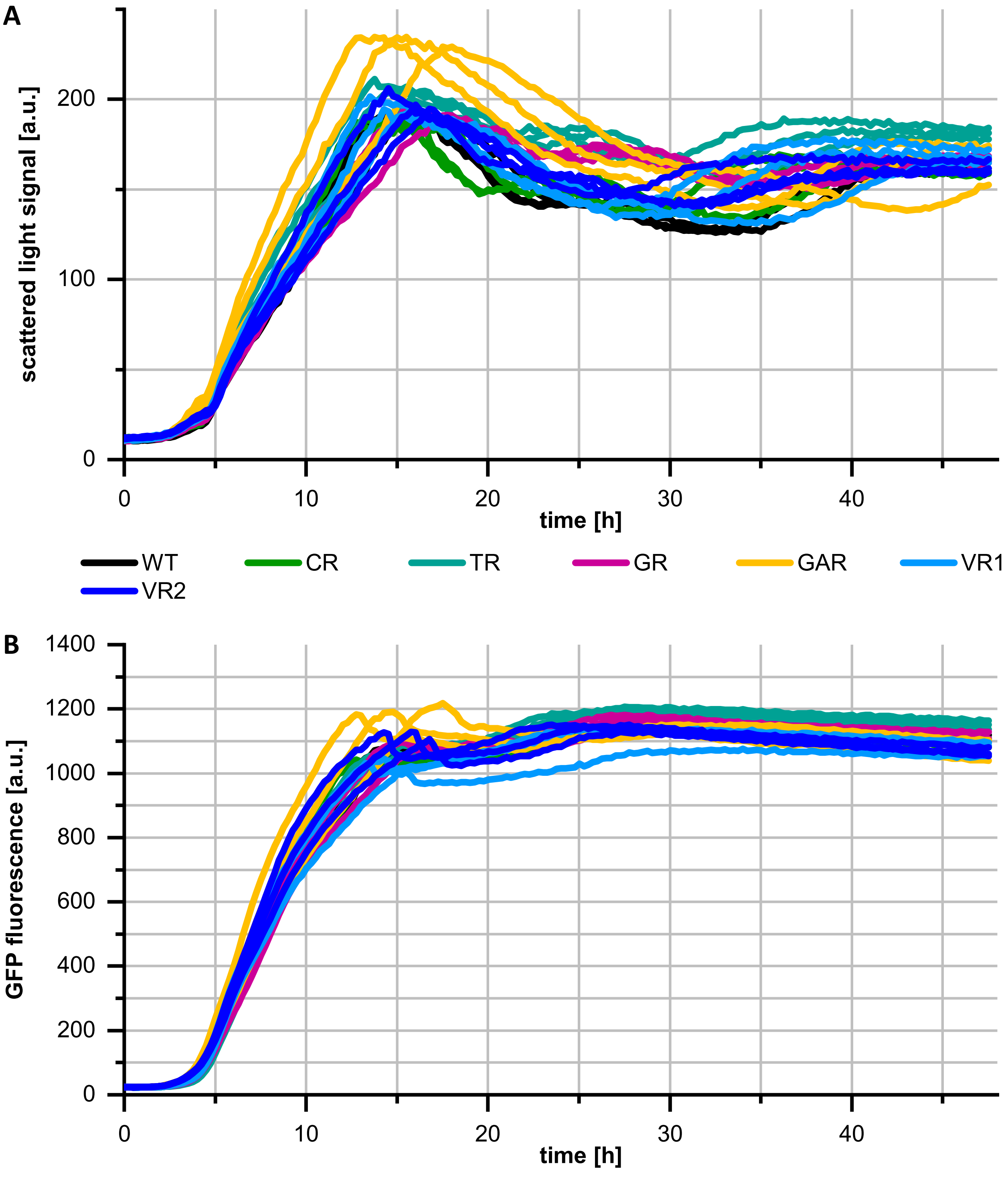

Figure S21. Growth of three different cultures of *P. putida* GS1 WT and mutants + pMiS4-eGFP without monoterpene or monoterpenoid. Tolerance assays were conducted in a microbioreactor system over 48 h. Biomass formation was monitored every 10 - 15 minutes via (A) scattered light signal intensity (absorbance at 620 nm) and (B) GFP fluorescence signal intensity (excitation filter: 488 nm; emission filter: 520 nm).

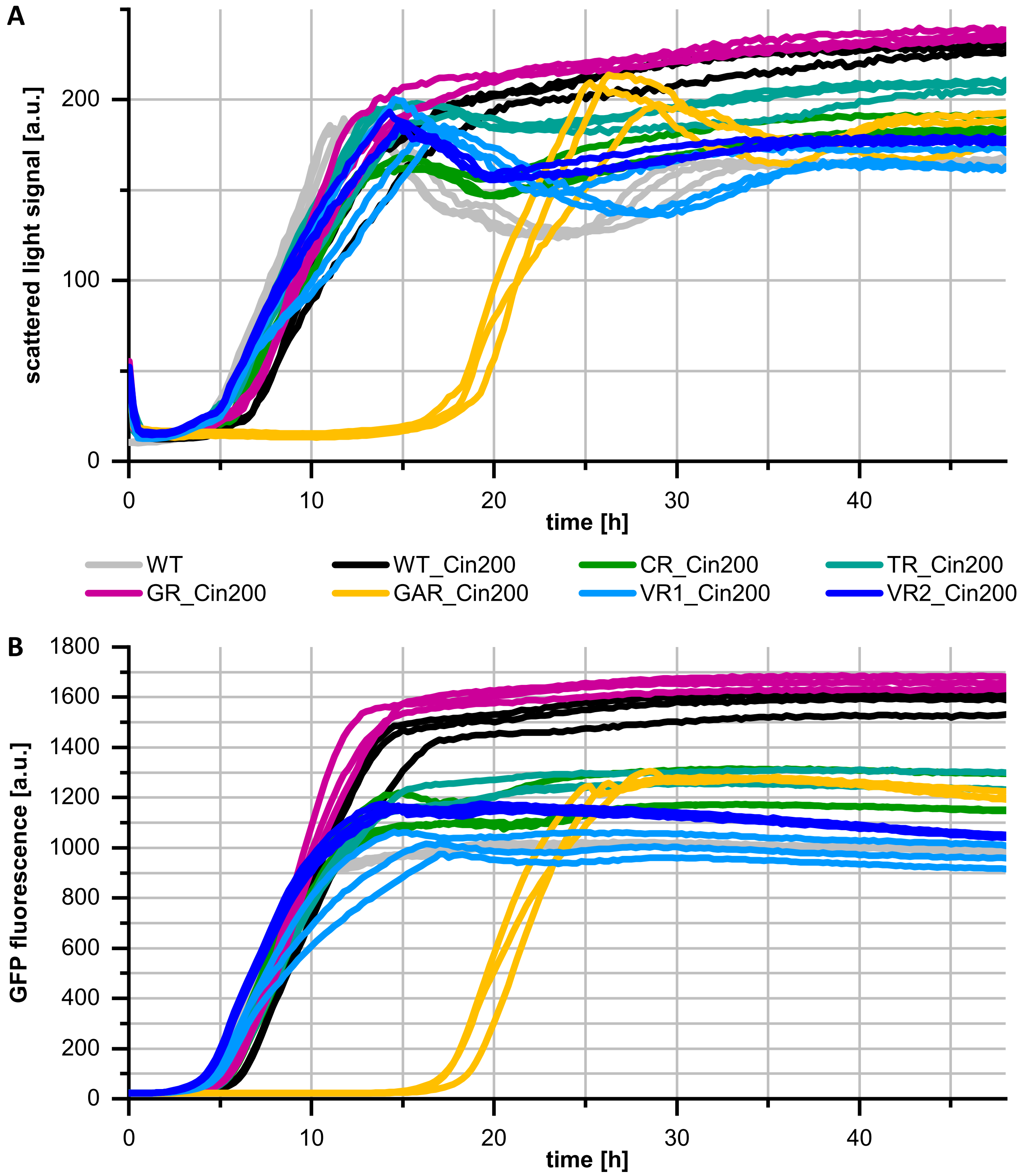

Figure S22. Growth of three different cultures of *P. putida* GS1 WT and mutants + pMiS4-eGFP without and in the presence of 200 mM 1,8-cineole. Tolerance assays were conducted in a microbioreactor system over 48 h. Biomass formation was monitored every 10 - 15 minutes via (A) scattered light signal intensity (absorbance at 620 nm) and (B) GFP fluorescence signal intensity (excitation filter: 488 nm; emission filter: 520 nm).

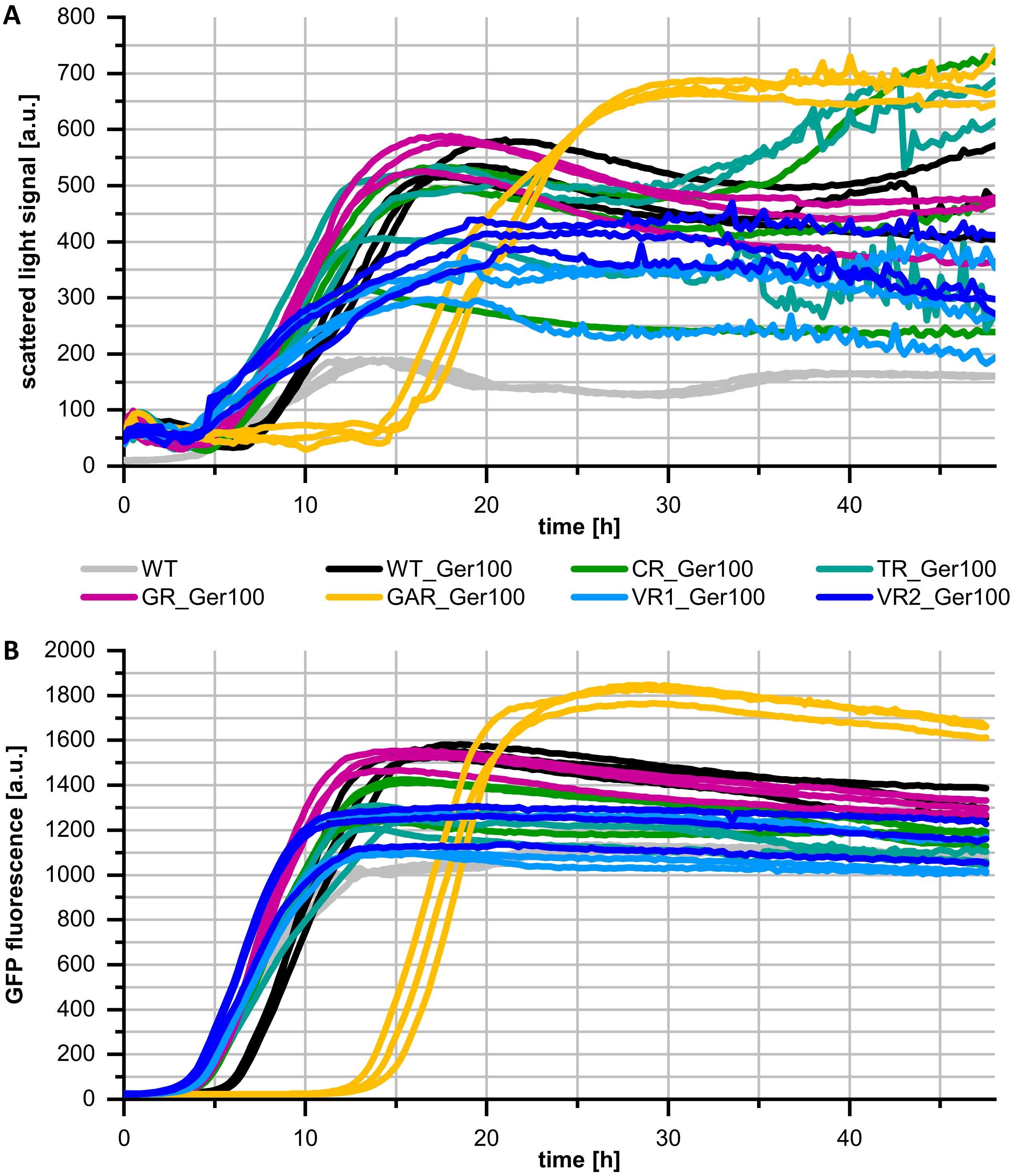

Figure S23. Growth of three different cultures of *P. putida* GS1 WT and mutants + pMiS4-eGFP without and in the presence of 100 mM geraniol. Tolerance assays were conducted in a microbioreactor system over 48 h. Biomass formation was monitored every 10 - 15 minutes via (A) scattered light signal intensity (absorbance at 620 nm) and (B) GFP fluorescence signal intensity (excitation filter: 488 nm; emission filter: 520 nm).

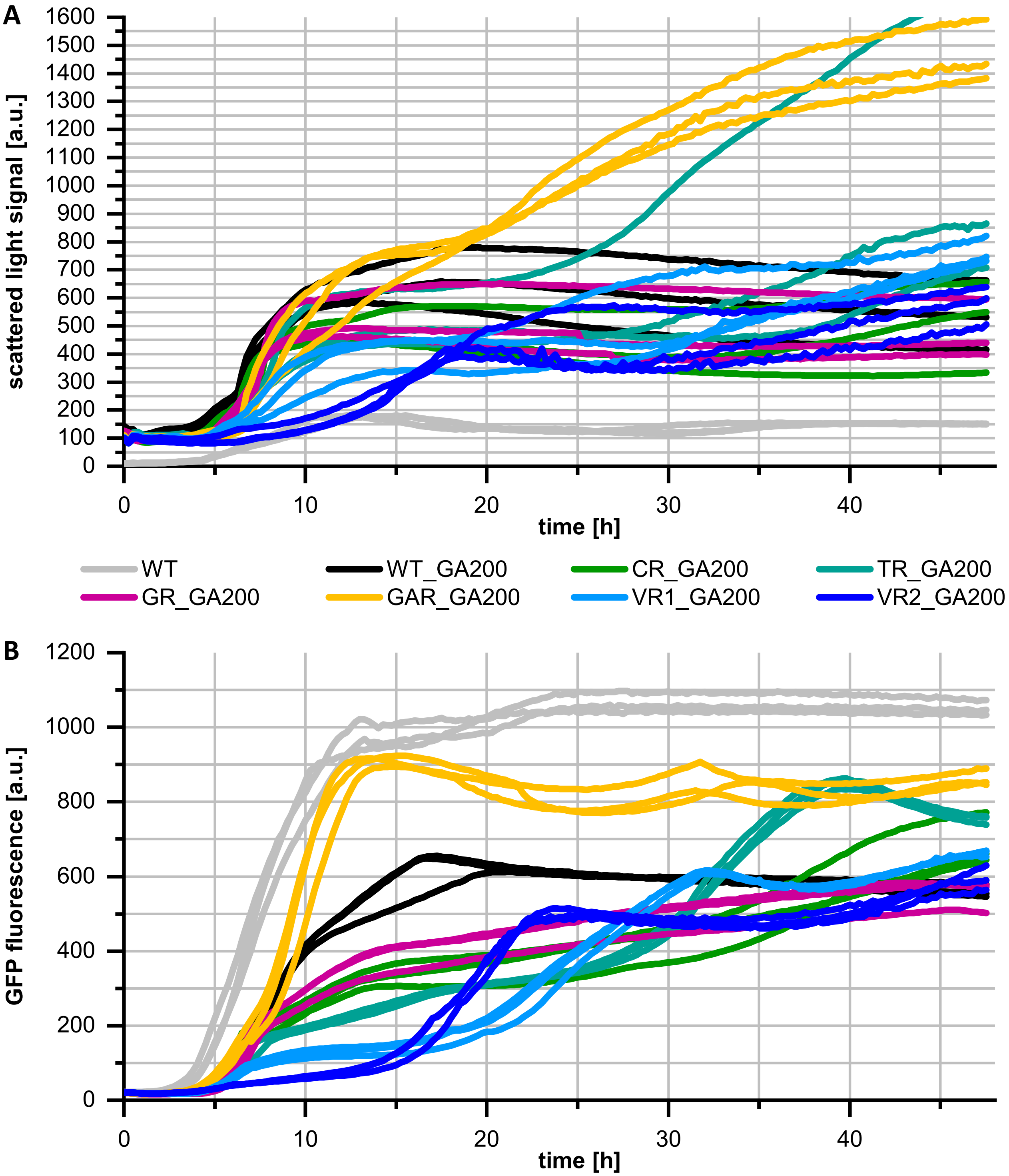

Figure S24. Growth of three different cultures of *P. putida* GS1 WT and mutants + pMiS4-eGFP without and in the presence of 200 mM geranic acid. Tolerance assays were conducted in a microbioreactor system over 48 h. Biomass formation was monitored every 10 - 15 minutes via (A) scattered light signal intensity (absorbance at 620 nm) and (B) GFP fluorescence signal intensity (excitation filter: 488 nm; emission filter: 520 nm).

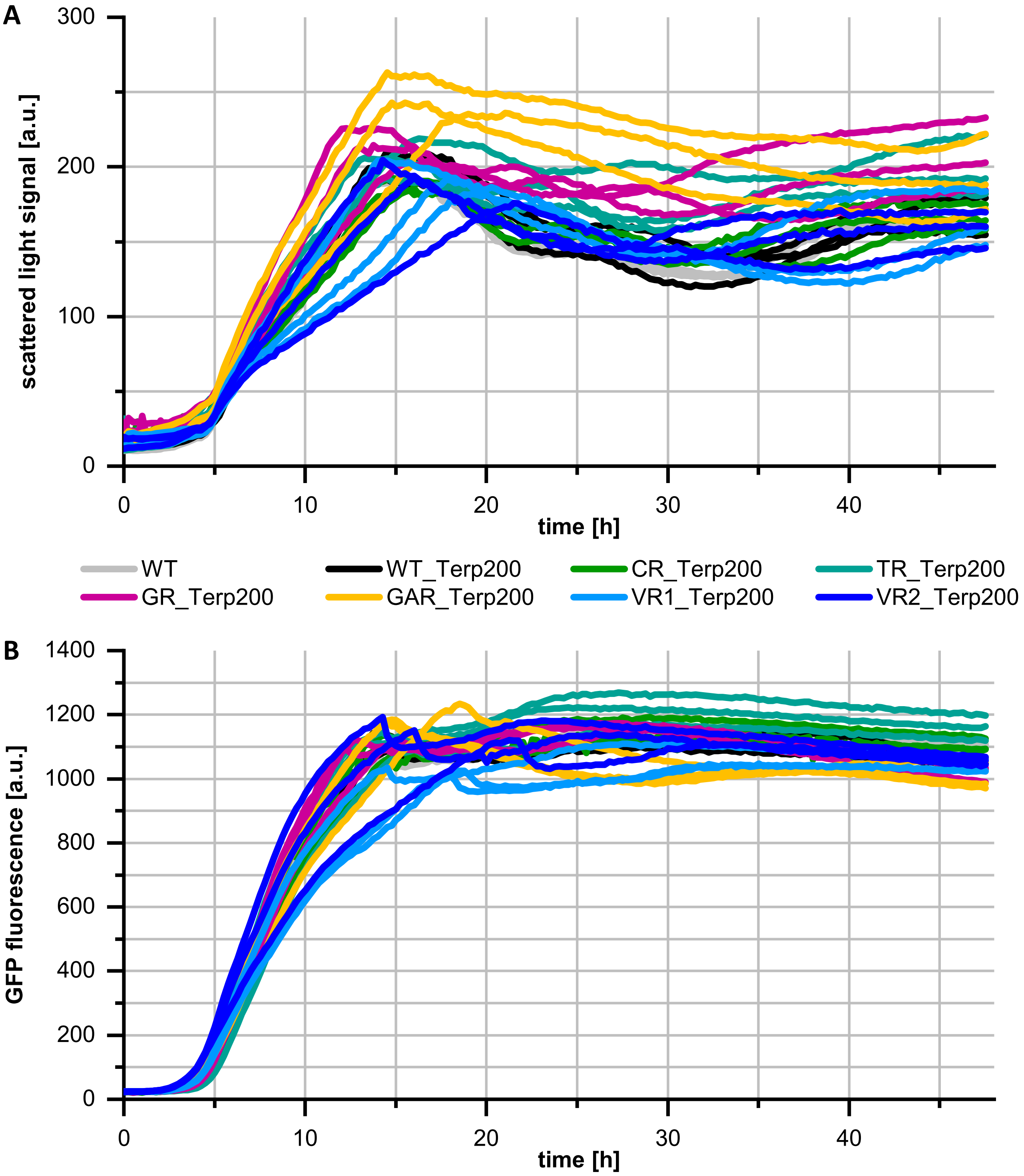

Figure S25. Growth of three different cultures of *P. putida* GS1 WT and mutants + pMiS4-eGFP without and in the presence of 200 mM γ-terpinene. Tolerance assays were conducted in a microbioreactor system over 48 h. Biomass formation was monitored every 10 - 15 minutes via (A) scattered light signal intensity (absorbance at 620 nm) and (B) GFP fluorescence signal intensity (excitation filter: 488 nm; emission filter: 520 nm).

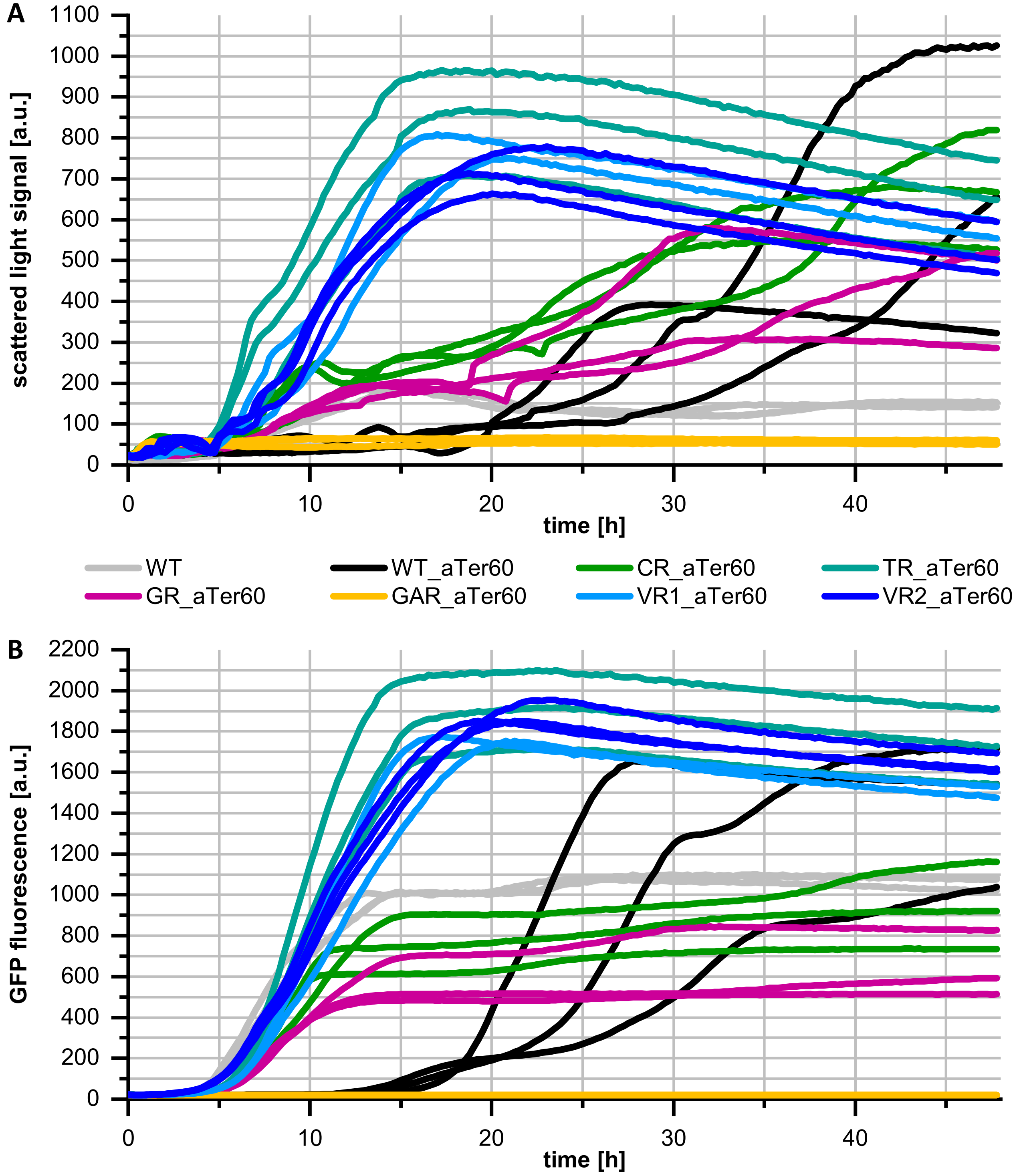

Figure S26. Growth of two (VR1) or three different cultures of *P. putida* GS1 WT and mutants + pMiS4-eGFP without and in the presence of 60 mM α-terpineol. Tolerance assays were conducted in a microbioreactor system over 48 h. Biomass formation was monitored every 10 - 15 minutes via (A) scattered light signal intensity (absorbance at 620 nm) and (B) GFP fluorescence signal intensity (excitation filter: 488 nm; emission filter: 520 nm).

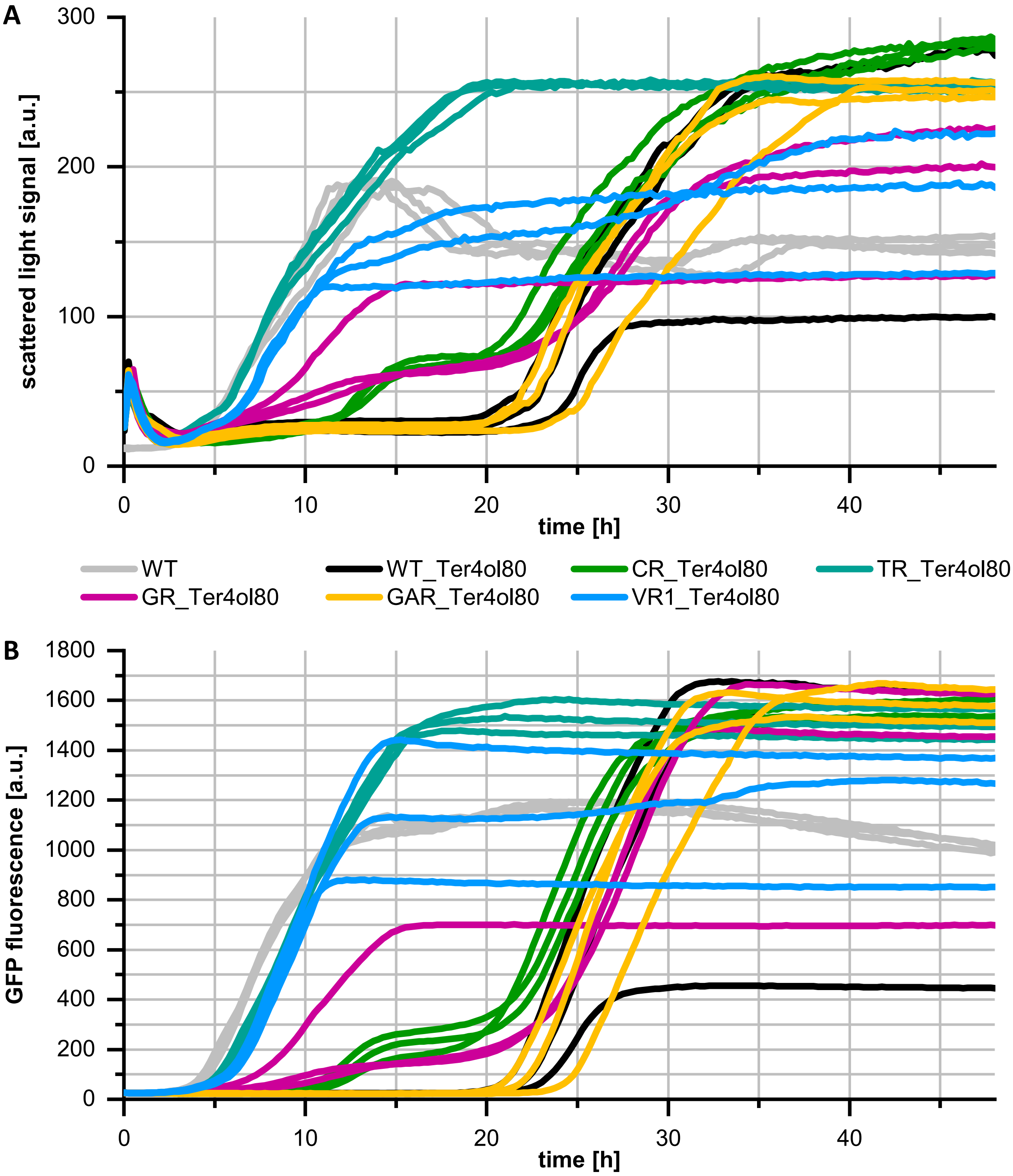

Figure S27. Growth of three different cultures of *P. putida* GS1 WT and mutants + pMiS4-eGFP without and in the presence of 80 mM terpinen-4-ol. Tolerance assays were conducted in a microbioreactor system over 48 h. Biomass formation was monitored every 10 - 15 minutes via (A) scattered light signal intensity (absorbance at 620 nm) and (B) GFP fluorescence signal intensity (excitation filter: 488 nm; emission filter: 520 nm).

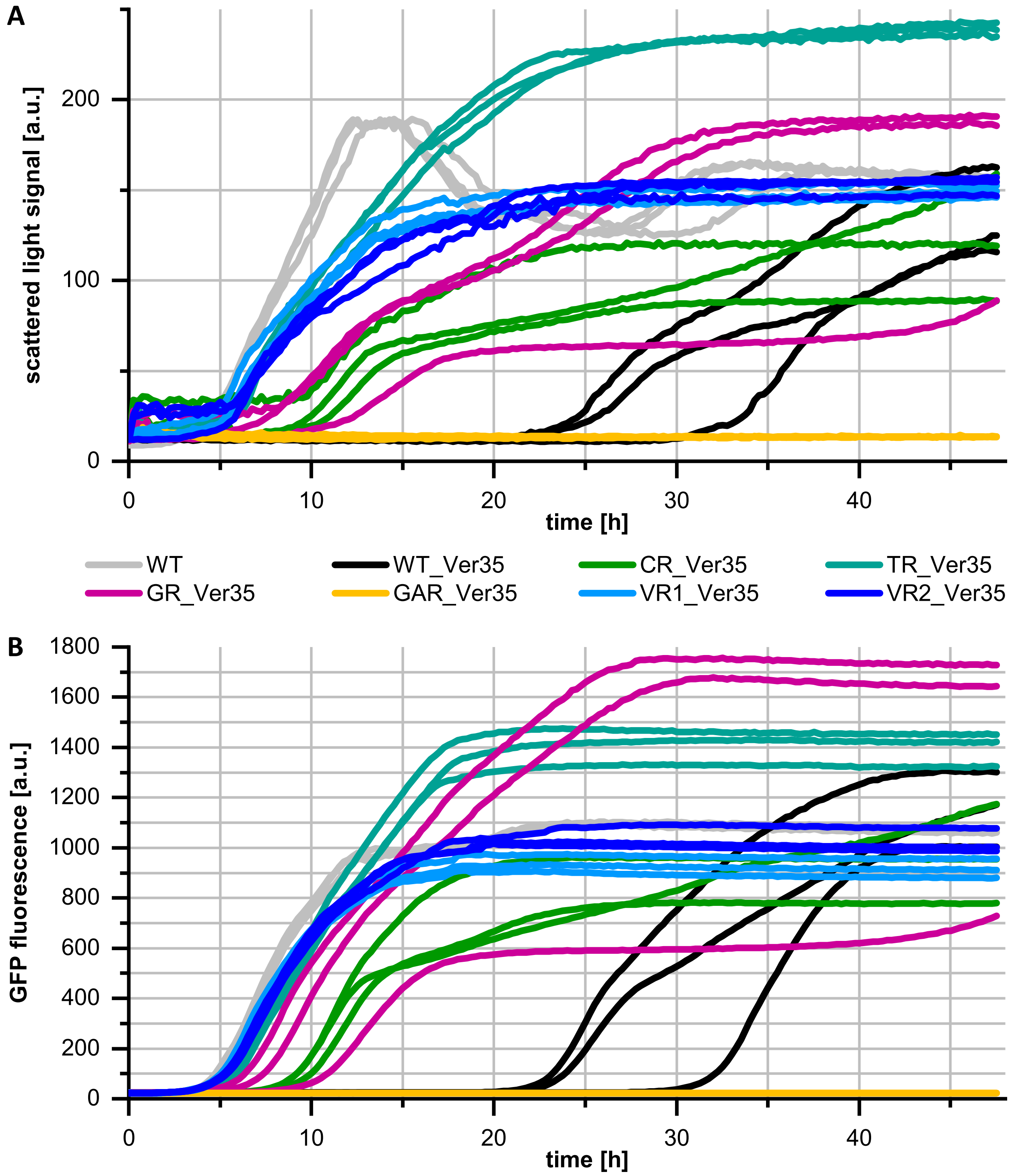

Figure S28. Growth of three different cultures of *P. putida* GS1 WT and mutants + pMiS4-eGFP without and in the presence of 35 mM verbenone. Tolerance assays were conducted in a microbioreactor system over 48 h. Biomass formation was monitored every 10 - 15 minutes via (A) scattered light signal intensity (absorbance at 620 nm) and (B) GFP fluorescence signal intensity (excitation filter: 488 nm; emission filter: 520 nm).

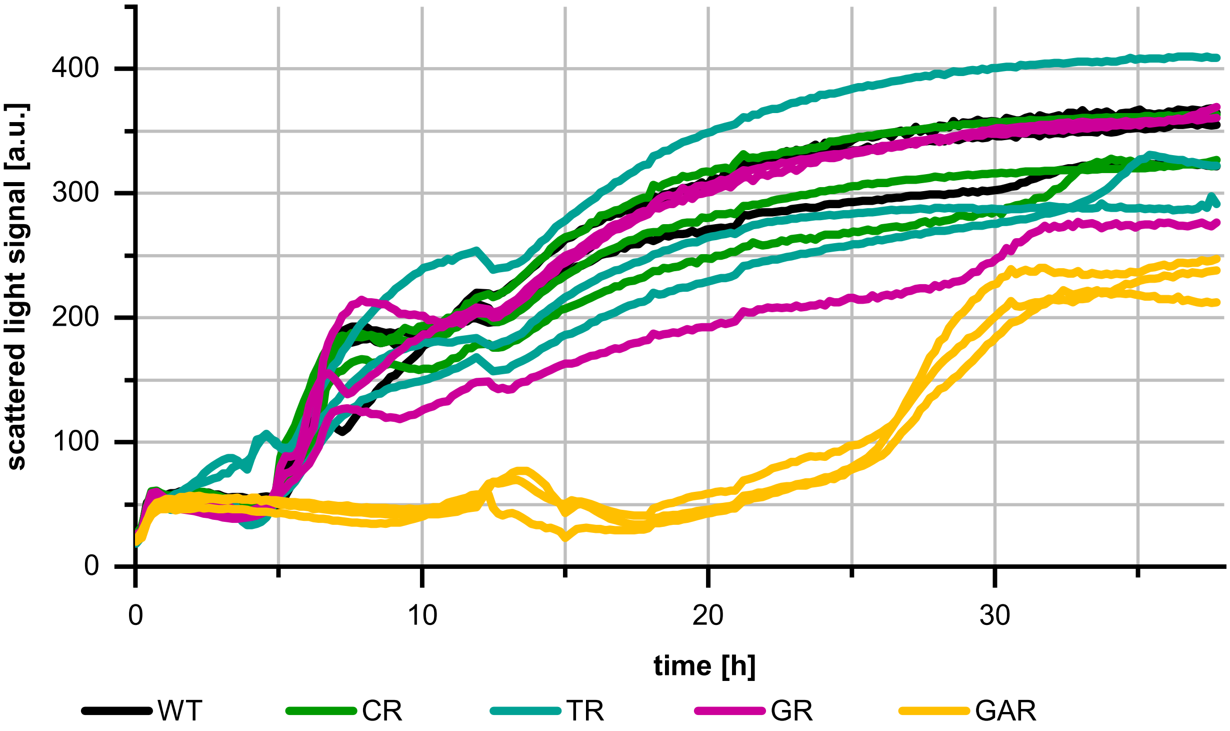

Figure S29. Growth of three different cultures of *P. putida* GS1 WT and mutants (CR, TR, GAR) in a geraniol to geranic acid biotransformation. The experiment was conducted in a microbioreactor system over 38 h. To determine geranic acid concentration samples were taken at time points t = 0, 12, 21 and 38 h and analyzed via HPLC-MS. Biomass formation was monitored every 10 - 15 minutes via scattered light signal intensity (absorbance at 620 nm).

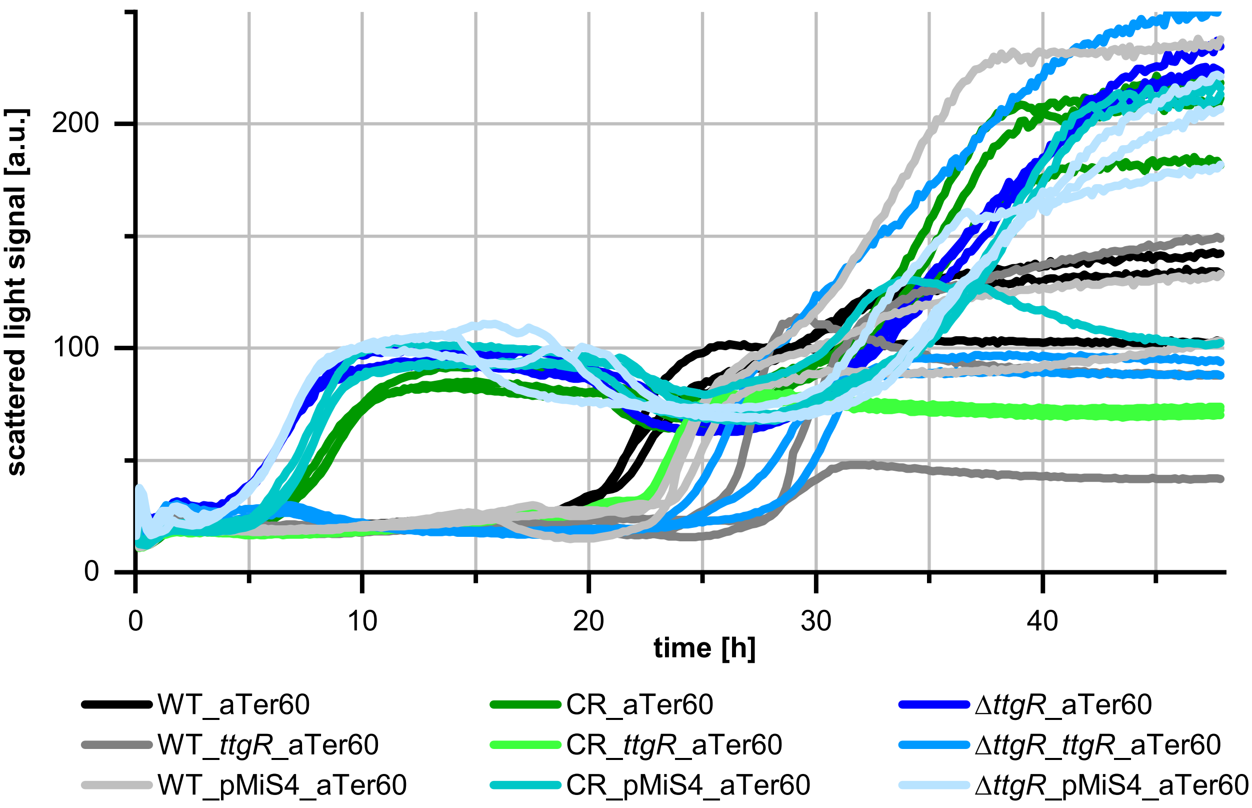

Figure S30. Verification of *ttgR* involvement in α-terpineol-hypertolerance phenotype by deletion and complementation. Growth curves of three individual cultures with 60 mM α-terpineol are shown. _pMiS4_ = strains contain the pMiS4 empty plasmid, _*ttgR*_ = strains contain the pMiS4-*ttgR* vector. Tolerance assays were conducted in a microbioreactor system over 48 h. Biomass formation was monitored every 10 - 15 minutes via (A) scattered light signal intensity (absorbance at 620 nm) and (B) GFP fluorescence signal intensity (excitation filter: 488 nm; emission filter: 520 nm).

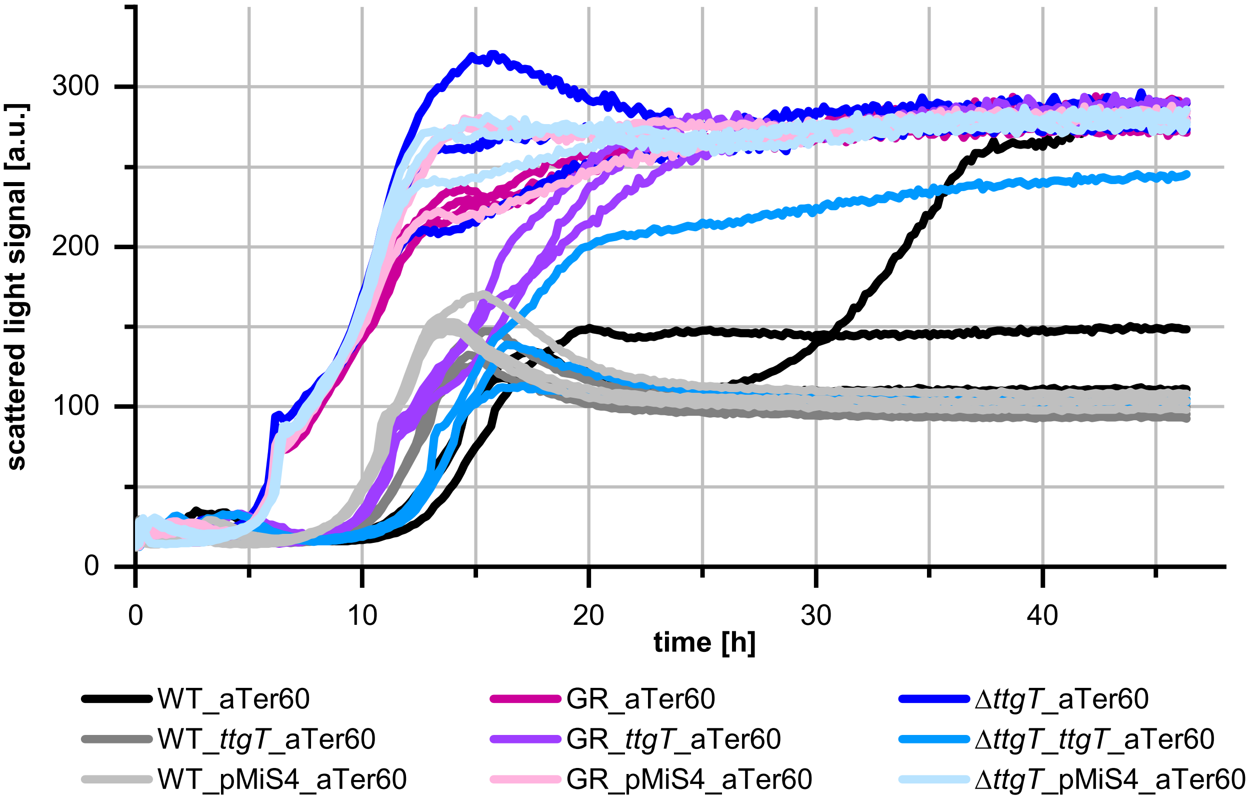

Figure S31. Verification of *ttgT* involvement in α-terpineol-hypertolerance phenotype by deletion and complementation. Growth curves of three individual cultures with 60 mM α-terpineol are shown. _pMiS4_ = strains contain the pMiS4 empty plasmid, _*ttgT*_ = strains contain the pMiS4-*ttgT* vector. Tolerance assays were conducted in a microbioreactor system over 48 h. Biomass formation was monitored every 10 - 15 minutes via (A) scattered light signal intensity (absorbance at 620 nm) and (B) GFP fluorescence signal intensity (excitation filter: 488 nm; emission filter: 520 nm).

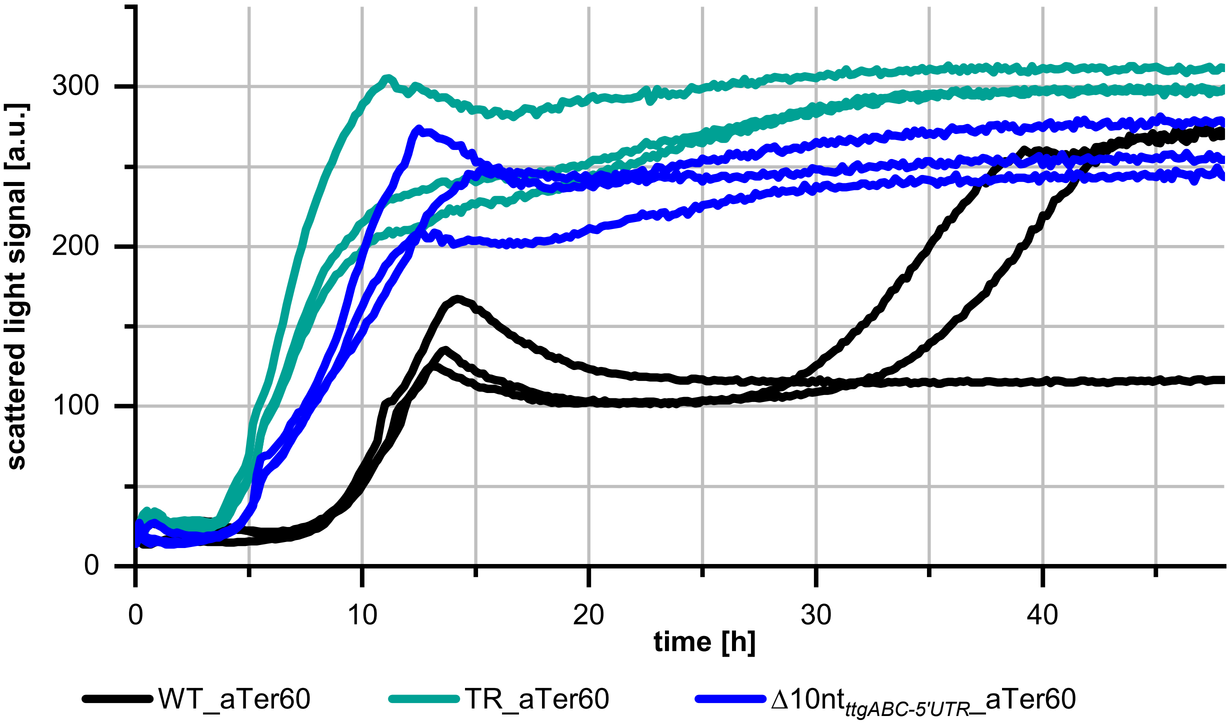

Figure S32. Verification of Δ10nt$_{ttgABC\text{-}5'UTR}$ involvement in α-terpineol-hypertolerance phenotype by deletion. Growth curves of three individual cultures with 60 mM α-terpineol are shown. Tolerance assays were conducted in a microbioreactor system over 48 h. Biomass formation was monitored every 10 - 15 minutes via (A) scattered light signal intensity (absorbance at 620 nm) and (B) GFP fluorescence signal intensity (excitation filter: 488 nm; emission filter: 520 nm).

References – Supplementary material

Franden MA, Jayakody LN, Li WJ, Wagner NJ, Cleveland NS, Michener WE, Hauer B, Blank LM, Wierckx N, Klebensberger J, Beckham GT (2018) Engineering *Pseudomonas putida* KT2440 for efficient ethylene glycol utilization. Metab Eng 48:197–207 . https://doi.org/10.1016/j.ymben.2018.06.003

Grant SG, Jessee J, Bloom FR, Hanahan D (1990) Differential plasmid rescue from transgenic mouse DNAs into *Escherichia coli* methylation-restriction mutants. Proc Natl Acad Sci 87:4645–4649 . https://doi.org/10.1073/pnas.87.12.4645

Hanahan D (1985) Techniques for transformation of *E. coli*. In: Glover DM (ed) DNA cloning: a practical approach, 3. IRL Press, Oxford, United Kingdom, United Kingdom, pp 109–135

Klebensberger J, Lautenschlager K, Bressler D, Wingender J, Philipp B (2007) Detergent-induced cell aggregation in subpopulations of *Pseudomonas aeruginosa* as a preadaptive survival strategy. Environ Microbiol 9:2247–2259 . https://doi.org/10.1111/j.1462-2920.2007.01339.x

Martínez-García E, de Lorenzo V (2011) Engineering multiple genomic deletions in Gram-negative bacteria: analysis of the multi-resistant antibiotic profile of *Pseudomonas putida* KT2440. Environ Microbiol 13:2702–2716 . https://doi.org/10.1111/j.1462-2920.2011.02538.x

Mi J, Sydow A, Schempp F, Becher D, Schewe H, Schrader J, Buchhaupt M (2016) Investigation of plasmid-induced growth defect in *Pseudomonas putida*. J Biotechnol 231:167–173 . https://doi.org/10.1016/j.jbiotec.2016.06.001

Simon R, Priefer U, Pühler A (1983) A broad host range mobilization system for in vivo genetic engineering: Transposon mutagenesis in Gram-negative bacteria. Bio/Technology 1:784–791 . https://doi.org/10.1038/nbt1183-784

Speelmans G, Bijlsma A, Eggink G (1998) Limonene bioconversion to high concentrations of a single and stable product, perillic acid, by a solvent-resistant *Pseudomonas putida* strain. Appl Microbiol Biotechnol 50:538–544 . https://doi.org/10.1007/s002530051331
